# Anti-cancer strategy targeting the energy metabolism of tumor cells surviving a low-nutrient acidic microenvironment

**DOI:** 10.1016/j.molmet.2020.101093

**Published:** 2020-09-30

**Authors:** Yuki Maeda, Ryota Kikuchi, Junichiro Kawagoe, Takao Tsuji, Nobuyuki Koyama, Kazuhiro Yamaguchi, Hiroyuki Nakamura, Kazutetsu Aoshiba

**Affiliations:** 1 Department of Respiratory Medicine, Tokyo Medical University Ibaraki Medical Center, 3-20-1 Chuou, Ami-machi, Inashiki-gun, Ibaraki, 300-0395, Japan; 2 Department of Respiratory Medicine, Tokyo Medical University, 6-7-1 Nishishinjuku, Shinjuku-ku, Tokyo, 160-0023, Japan; 3 Department of Medicine, Otsuki Municipal Hospital, 1255 Hanasaki, Otsuki-chou, Otsuki-shi, Yamanashi, 401-0015, Japan; 4 Department of Clinical Oncology, Tokyo Medical University Ibaraki Medical Center, 3-20-1 Chuou, Ami-machi, Inashiki-gun, Ibaraki, 300-0395, Japan

**Keywords:** Lung cancer, Glucose, Acidosis, Mitochondrion, Uncoupler

## Abstract

**Objective:**

Tumor cells experience hypoxia, acidosis, and hypoglycemia. Metabolic adaptation to glucose shortage is essential to maintain tumor cells’ survival because of their high glucose requirement. This study evaluated the hypothesis that acidosis might promote tumor survival during glucose shortage and if so, explored a novel drug targeting metabolic vulnerability to glucose shortage.

**Methods:**

Cell survival and bioenergetics metabolism were assessed in lung cancer cell lines. Our in-house small-molecule compounds were screened to identify those that kill cancer cells under low-glucose conditions. Cytotoxicity against non-cancerous cells was also assessed. Tumor growth was evaluated in vivo using a mouse engraft model.

**Results:**

Acidosis limited the cellular consumption of glucose and ATP, causing tumor cells to enter a metabolically dormant but energetically economic state, which promoted tumor cell survival during glucose deficiency. We identified ESI-09, a previously known exchange protein directly activated by cAMP (EAPC) inhibitor, as an anti-cancer compound that inhibited cancer cells under low-glucose conditions even when associated with acidosis. Bioenergetic studies showed that independent of EPAC inhibition, ESI-09 was a safer mitochondrial uncoupler than a classical uncoupler and created a futile cycle of mitochondrial respiration, leading to decreased ATP production, increased ATP dissipation, and fuel scavenging. Accordingly, ESI-09 exhibited more cytotoxic effects under low-glucose conditions than under normal glucose conditions. ESI-09 was also more effective than actively proliferating cells on quiescent glucose-restricted cells. Cisplatin showed opposite effects. ESI-09 inhibited tumor growth in lung cancer engraft mice.

**Conclusions:**

This study highlights the acidosis-induced promotion of tumor survival during glucose shortage and demonstrates that ESI-09 is a novel potent anti-cancer mitochondrial uncoupler that targets a metabolic vulnerability to glucose shortage even when associated with acidosis. The higher cytotoxicity under lower-than-normal glucose conditions suggests that ESI-09 is safer than conventional chemotherapy, can target the metabolic vulnerability of tumor cells to low-glucose stress, and is applicable to many cancer cell types.

## Highlights

• Tumor cells experience hypoxia, acidosis, and glucose shortage.
• Tumor acidosis promotes metabolic adaptation to glucose shortage.
• ESI-09, a known EAPC inhibitor, is a novel and safe mitochondrial uncoupler.
• ESI-09 kills tumor cells during glucose shortage, even under acidosis conditions.
• ES-09 targets metabolically dormant and chemotherapy-resistant tumor cells.

## 1 Introduction

Tumor cells face environmental stresses, such as hypoxia, acidosis, and a shortage of nutrients, caused by high demands of nutrients and waste removal, but they also have an insufficient vascular network [1]. The best studied stress in the tumor microenvironment (TME) is hypoxia, which often reaches as low as a pO_2_ level of 1 mmHg because a large amount of O_2_ is consumed by rapidly proliferating tumor cells and disorganized vascular networks increase the O_2_ diffusion distance [2]. Acidosis is another environmental stress caused by the accumulation of tumor-derived acid, such as lactate, H^+^, and CO_2_; increased H^+^ efflux via membrane-associated H^+^ transporters, such as H^+^-ATPase, Na^+^-H^+^ exchanger, and HCO_3_^−^ exchanger, in association with enhanced extracellular hydration of CO_2_ by membrane-bound carbonic anhydrase 9; and reduced elimination of tumor-derived acid due to poor perfusion [3]. Decreased extracellular pH of tumors, which reportedly reaches as low as 6.5 to 6.9 [[3], [4], [5], [6], [7]], is thought to induce tumor progression, invasion, chemoresistance, impaired immune surveillance, and metabolic reprograming [[8], [9], [10]].

The third critical stress in the TME is nutrient deficiency, which is caused by increased nutrient demand for rapid tumor proliferation not met by an adequate supply [11,12]. Although the TME has deficiencies in various nutrients, glucose deprivation is the most common one because of the high rate of glucose consumption by tumor cells, so-called “aerobic glycolysis” [[13], [14], [15]], which often reaches a value of <1 mM within solid tumor masses [4,12]. To cope with glucose deprivation, tumor cells utilize lactate, glutamine, and fatty acids as alternative fuel sources [1,16], degrade macromolecules from their own components (autophagy) [17] or the microenvironment [12], and synthesize glucose from lactate and amino acids [18].

Although acidosis has been shown to induce a stress response similar to a low nutritional stress response, the role of acidosis in metabolic adaptation to nutrient limitations is not well understood [[19], [20], [21], [22], [23]]. We hypothesized that acidosis might promote tumor cell survival during glucose shortage. We investigated the role of acidosis in promoting tumor cell survival under low-glucose conditions. We found that acidosis reprograms tumor cell metabolism to efficiently save energy (ATP) and fuel (glucose), causing tumor cells to enter a metabolically dormant but energetically economic state that supports tumor cell survival under glucose shortage conditions. We describe a promising new anti-cancer therapy using ESI-09, a previously known inhibitor of exchange protein directly activated by cAMP (EPAC), which acts as a potent mitochondrial uncoupler independent of EPAC inhibition and targets metabolic vulnerability to low-glucose conditions even when associated with acidosis. Our study results provide a better understanding of the role of the mitochondrial uncoupler in cancer therapy.

## 2 Materals and methods

### 2.1 Reagents

The reagents used in this study are listed in Supplementary Table S1.

### 2.2 Cell culture

Non-small cell lung cancer cell lines A549 (CCL-185), H1299 (CRL-5803), and H1975 cells (CRL-5908) were obtained from the American Type Culture Collection (ATCC, Manassas, VA, USA). PC3 cells (JCRB0077), another non-small cell lung cancer cell line, were obtained from the Japanese Collection of Research Bioresources Cell Bank (Osaka, Japan). The cell lines were authenticated by short tandem repeat profiling using a Promega PowerPlex 16 HS system (Promega Corporation, Madison, WI, USA). Human fetal lung IMR-90 (CCL-186) fibroblasts were obtained from ATCC. The A549 and IMR-90 cells were maintained in Dulbecco's Modified Eagle medium (DMEM, Gibco; Thermo Fisher Scientific, Inc., Waltham, MA, USA) containing 10% fetal bovine serum (FBS) at 37 °C in a humidified incubator saturated with a gas mixture containing 5% CO_2_. The H1299, H1975, and PC3 cells were maintained in Roswell Park Memorial Institute (RPMI) 1640 medium containing 10% FBS. Human pulmonary alveolar epithelial cells (#3200) were obtained from ScienCell Research Laboratories, Inc. (San Diego, CA, USA) and grown in AEPiCM medium (#3201; ScienCell). Human lung microvascular endothelial cells (cc-2527) were obtained from Cambrex Bio Science Walkersville (Walkersville, MD, USA) and grown in a EGM-2MV Bullet kit medium (cc-3202; ScienCell). To assess cell survival, confluent cells were serum-starved for 48 h in medium containing 0.5% FBS and then incubated in DMEM adjusted to pH 7.4 or pH 6.8 by adding a 4-(2-hydroxyethyl)-1-piperazineethanesulfonic acid (HEPES, 50 mM) solution and an HCl solution supplemented with 0.5% FBS and different glucose concentrations in the presence or absence of the tested reagents. When media were not treated with an HCl solution, an equiosmolar amount of NaCl was included to eliminate any potential effect of differences in medium osmolality. The cell number was measured as described to follow. To assess cell proliferation, actively dividing cells in growth medium containing 10% FBS were monitored for cell number.

### 2.3 Measurement of cell number

Cell number was evaluated using a Hoechst 33,342 DNA quantification assay. Briefly, cells in a 96-well culture plate were lysed in 100 μl of distilled water, followed by a freeze-thaw cycle. The cell lysates then were solubilized in 100 μl of TNE buffer (10 mM Tris, 1 mM EDTA, 2 M NaCl, and pH 7.4) containing 10 μg/ml of Hoechst 33,342 (#14533; Sigma–Aldrich Japan, Tokyo, Japan). The fluorescence intensities were read at an excitation wavelength of 350 nm and emission wavelength of 460 nm using a microplate fluorometer (PerkinElmer Arvo X2; PerkinElmer Japan Co., Ltd., Tokyo, Japan).

### 2.4 Determination of live, apoptotic, and necrotic cells

The live, apoptotic, and necrotic cells were distinguished by dual-fluorescence staining with ethidium bromide (2 μg/ml) and Hoechst 33,342 (2 μg/ml) [24]. Under microscopic examination, the live cells had normal nuclear morphology with blue fluorescence. The apoptotic cells had condensed or fragmented nuclei with blue (early stage apoptosis) or red (late-stage apoptosis) fluorescence. Cells with red nuclei with normal nuclear morphology (that is, with no signs of chromatin condensation or fragmentation) were scored as necrotic. A minimum of 300 cells was counted using an Olympus IX71 microscope (Olympus Optical Co., Ltd., Tokyo, Japan).

### 2.5 Lactate dehydrogenase (LDH) assay

LDH activity was evaluated using an enzymatic method with a Cytotoxicity LDH assay WST kit (#CK12; Dojindo Molecular Technologies, Inc., Kumamoto, Japan).

### 2.6 Depletion of mitochondrial (mt) DNA

A549 cells devoid of mtDNA (A549 ρ^0^) were prepared by culturing cells in DMEM supplemented with 10% FBS in the presence of ethidium bromide (50 ng/ml), sodium pyruvate (1 mM), and uridine (100 μg/ml) for > 20 generations. The control parental A549 cells were maintained for the same period in normal culture medium. The successful establishment of the A549 ρ^0^ cell line was confirmed by polymerase chain reactions using mitochondrial DNA-specific primers in our previous study [25].

### 2.7 In vitro wound-healing assay

The cells’ migratory activity was determined by an in vitro wound-healing assay [26]. Wound healing was observed through a phase-contrast Olympus IX71 microscope (Olympus Optical Co., Ltd.). The wound area was measured using image analysis software (Win Roof Version 3.5; Mitani Corporation, Fukui, Japan) on a Microsoft XP computer. Five measurements were taken from five fields in each well obtained from six wells in each experiment.

### 2.8 Measurement of intracellular ATP and ADP levels

Intracellular ATP contents were measured using Cellno ATP assay reagent (Toyo B-Net, Co., Ltd., Tokyo, Japan) according to the manufacturer's instructions. Briefly, 100 μl of lysis/assay solution provided by the manufacturer was added to confluent cell cultures in 96-well plates. After the plates were shaken for 1 min and incubated for 10 min at 23 °C, luminescence was measured in a microplate luminometer (PerkinElmer Arvo X2). The ADP/ATP ratio was determined using an EnzyLight ADP/ATP assay kit (BioAssay Systems, Hayward, CA, USA) according to the manufacturer's instructions.

### 2.9 Measurement of ATP consumption rate

Cells were incubated in DMEM containing 10 mM of glucose and 4 mM of glutamine at pH 7.4 or 6.8. Intracellular ATP contents were determined before and 10 min after the addition of glycolysis inhibitor 2-deoxyglucose (2-DG, 30 mM, and #040-06481; Fujifilm Wako Pure Chemical Corporation, Osaka, Japan) and mitochondrial ATP synthase inhibitor oligomycin A (2 μM). The decrease in intracellular ATP contents following the addition of oligomycin A and 2-DG was referred to as the rate of cellular ATP consumption. The proportion of ATP consumption by protein synthesis and DNA/ribosomal (rRNA) synthesis was determined by measuring the ATP consumption rate in the presence of cycloheximide (2.5 mM) or actinomycin D (8 μM), respectively.

### 2.10 Measurement of ATP consumption induced by mitochondrial F_1_F_0_ ATP hydrolase

Intracellular ATP contents were determined before and 10 min after the addition of 2-DG (30 mM), and the mitochondrial uncouplers were tested in the presence or absence of selective F_1_F_0_ ATP hydrolase inhibitor BMS-199264 (2 μM). Differences in intracellular ATP contents between the presence and absence of BMS-199264 were referred to as the amount of ATP consumption by mitochondrial F_1_F_0_ ATP hydrolase.

### 2.11 Measurements of glucose consumption, lactate production, ATP output, and allocation to glycolysis or OXPHOS

Confluent cells were incubated in 96-well plates at the indicated pH levels. After 24 h, a portion of medium from each well was removed and the glucose and lactate concentrations in the culture and original medium (not cultured with cells) were measured using an amperometric blood glucose sensor (Nipro Care Fast C; Nipro Co., Osaka, Japan) and an enzymatic method at an outsourcing laboratory (LSI Medience Co., Tokyo, Japan), respectively. Glucose consumption and lactate production were calculated based on the difference between the glucose or lactate concentrations in the original medium vs the culture medium. The relative glucose consumption and lactate production rates were calculated by normalizing each treatment to the control group. The amounts of glycolysis and OXPHOS components in the bioenergetic process were calculated using the following formula [27]: Lac_(c)_ = lactate concentration in the control medium after 6 h of incubation; Lac_(o)_ = lactate concentration in the medium after 6 h of incubation with 2 μM of oligomycin; glycolysis % = Lac_(c)_ × 100/Lac_(o)_; and OXPHOS % = 100 - glycolysis %. The amount of glucose used for glycolysis was calculated using the following formula based on the assumption that two lactate molecules are produced during glycolysis of one glucose molecule; glucose used for glycolysis (mol) = amount of lactate production (mol)/2.

### 2.12 Measurements of amino acid concentration

The amino acid content in the culture medium was measured by liquid chromatography-tandem mass spectrometry at SRL, Inc. (Tokyo, Japan).

### 2.13 In situ nuclear run-on assay

Ongoing rRNA synthesis was assessed by fluorouridine (FUrd) incorporation in in situ run-on assays [28]. Briefly, cells were labeled with 2 mM of FUrd (Tokyo Chemical Industry, Tokyo, Japan) for 20 min at 37 °C. The cells were then fixed with 10% formalin, permeabilized with methanol, and incubated with mouse anti-bromodeoxyuridine antibody (clone BU-31, #B2531, Sigma–Aldrich Japan), followed by incubation with Alexa Fluor 488-conjugated anti-mouse IgG antibody (#A-11001, Thermo Fisher Scientific). After counterstaining the cell nuclei with 4′,6-diamidino-2-phenylindole (DAPI), fluorescence was observed under an epifluorescence microscope, and the number of FUrd-incorporated cells out of 300 cells was quantified.

### 2.14 Surface sensing of translation (SUnSET) assay

Protein synthesis was assessed via the SUnSET technique using an anti-puromycin antibody for the immunological detection of puromycin peptides [29]. Briefly, cells were incubated with 2 μM of puromycin (Nakarai Tesque Inc., Kyoto, Japan) for 150 min at 37 °C and then lysed in radioimmunoprecipitation (RIPA) buffer. Equal concentrations of denatured protein lysates were resolved on 4%–10% sodium dodecyl sulfate polycacrylamide gel electrophoresis gels (4–10% Mini-Protean TGX gels; Bio-Rad laboratories, Inc., Hercules, CA, USA) and electrotransferred to polyvinylidene difluoride membranes, which were then incubated in 5% bovine serum albumin (BSA) in Tris-buffered saline at pH 7.4 with 0.05% Tween-20 (TBST) for 1 h at room temperature. The membranes were then probed with mouse monoclonal anti-puromycin antibody (#PEN-MA001, clone 3RH11; Cosmo Bio Co. Ltd., Tokyo Japan) in 5% BSA in TBST overnight at 4 °C. The membranes were washed with TBST and vigorous rocking at room temperature. The membranes were then incubated with anti-mouse immunoglobulin G conjugated with horseradish peroxidase in 5% BSA in TBST for 1 h at room temperature. The membranes were washed again and proteins were detected with chemiluminescence (SuperSignal West Pico, Thermo Fisher Scientific K.K., Tokyo, Japan).

### 2.15 Measurements of oxygen consumption and extracellular acidification

The oxygen consumption rate (OCR) and extracellular acidification rate (ECAR) were measured using a Seahorse XFp analyzer (Agilent, Santa Clara, CA, USA). The cellular bioenergetic profiles were measured using an XFp Cell Mito Stress test kit and XFp Mito Fuel Flex test kit (Agilent) according to the manufacturer's instructions [30].

### 2.16 Measurements of tricarboxylic acid (TCA) cycle activity

TCA cycle activity was assessed using media without glucose but containing 10 mM of galactose and 4 mM of glutamine to measure the ECAR [31]. Under this condition, the ECAR does not represent the glycolytic activity but the CO_2_ production in the mitochondrial matrix [31]. The TCA activity in the presence of glucose was assessed using an XFp Glycolytic Rate assay kit (Agilent) according to the manufacturer's instructions to measure mitochondria-associated acidification. The contribution of mitochondria-derived CO_2_ and glycolysis-derived lactate to extracellular acidification can be separately determined using this kit.

### 2.17 Mitochondrial respiration on selected substrates

The A549 cells were permeabilized with Seahorse XF Plasma Membrane Permeabilizer (1 nM, #102504-100; Agilent), and the mitochondria were stimulated with substrates for respiratory chain complex I (10 mM of pyruvate and 1 mM of malate), complex II/III (10 mM of succinate and 2 μM of rotenone), and complex IV (10 mM of ascorbate, 100 μM of TMPD, and 2 μM of antimycin A) in the presence of ADP (4 mM) in a mitochondrial assay solution (220 mM of mannitol, 70 mM of sucrose, 10 mM of KH_2_PO_4_, 5 mM of MgCl_2_, 2 mM of HEPES, 1 mM of EGTA, and 0.2% fatty acid-free BSA [32]. OCR was measured using a Seahorse XF analyzer.

### 2.18 Mitochondrial swelling assay

The protonophoric ability was measured by a proton-dependent mitochondrial swelling assay as previously described [33,34]. Briefly, mitochondria were isolated from the A549 cells by fractional centrifugation using a Mitochondria Isolation kit (#KC010100; BioChain Institute Inc., Newark, CA, USA). The isolated mitochondria (0.5 mg of mitochondrial protein) were suspended in 1 ml of isotonic acetate buffer (145 mM of potassium acetate, 5 mM of Tris–HCl, 0.5 mM of EDTA, 3 μM of valinomycin, 1 of μM rotenone, and pH 7.4). Mitochondrial swelling was recorded as the mitochondrial suspension's decrease in absorbance at 600 nm.

### 2.19 Assessment of mitochondrial membrane potential

Cells were loaded with the mitochondrial potential indicator tetramethyl rhodamine methyl ester (250 nM, #T688; Thermo Fisher Scientific, Yokohama, Japan) for 30 min at 37 °C, and fluorescence images were acquired using an Olympus IX71 microscope (Olympus Optical Co., Ltd.).

### 2.20 Assessment of plasma membrane potential

Cells were loaded with the plasma membrane potential indicator DiBAC4(3) (1 μM, #AS-84700; Anaspec, Inc., Fremont, CA, USA), and the fluorescence intensities were read using a microplate fluorometer at excitation/emission wavelengths of 485–530 nm.

### 2.21 Determination of intracellular nicotinamide adenine dinucleotide phosphate (NAD(P)H)

Intracellular NAD(P)H levels were measured by autofluorescence at excitation/emission wavelengths of 355–460 nm [35].

### 2.22 Oxygen concentration in culture medium from the closed culture system

The oxygen concentration in culture medium was determined using oxygen-sensor microplates (OxoPlate OP96C; PreSense Precision Sensing GmbH, Regensburg, Germany) according to the manufacturer's instructions. The well in the OxoPlate was overlaid with 300 μl of mineral oil to prevent diffusion of O_2_ in the medium into the air during the experiment.

### 2.23 Detection of hypoxia inside spheroids

Hypoxia inside the spheroids was detected using a LOX-1 hypoxia probe (#NC-LOX1S; Medical & Biological Laboratories Co., Ltd., Nagoya, Japan) according to the manufacturer's instructions. The hypoxic regions emitting red phosphorescence were visually detected through an Olympus IX71 microscope (Olympus Optical Co., Ltd.).

### 2.24 Xenograft model

All of the mice used in the experiments were cared for in accordance with the standards of the Institutional Animal Care and Use Committee under a protocol approved by the Animal Care and Use Committee of the CMIC Pharma Science Co., Ltd., Bioresearch Center (Yamanashi, Japan). Female BALB/c-nu (nu/nu) mice (5 weeks old) were obtained from Charles River Laboratories Japan, Inc. (Yokohama, Japan). The mice were kept in a 12-h light and dark cycle and acclimatized for 1 week before the study. To generate tumor xenografts, A549 cells (5 × 10^6^ cells) in 100 μl of phosphate-buffered saline were injected into the subcutaneous flanks of nude mice. Once tumors became palpable, the tumor size was measured twice weekly using calipers, and the tumor volume was calculated according to the formula: V = ab^2^/2, where a and b are the tumor length and width, respectively. When the tumor volumes reached an average of approximately 100–150 mm^3^, the mice were randomly assigned to one of the following six treatment groups: 1) vehicle (n = 6), 2) ESI-09 (2 mg/kg, daily, i.p., n = 7), 3) ESI-09 (10 mg/kg, daily, i.p., n = 7), 4) bevacizumab (5 mg/kg, twice weekly, i.p., n = 7), 5) bevacizumab (5 mg/kg, twice weekly, i.p.), and ESI-09 (2 mg/kg, daily, i.p.) (n = 7) or 6) bevacizumab (5 mg/kg, twice weekly, i.p.) and ESI-09 (10 mg/kg, daily, i.p.) (n = 7) for the indicated periods. At the end of the treatment period, blood samples were obtained by vena cava puncture under terminal anesthesia and the tumors were harvested, weighed, fixed in formalin, and embedded in paraffin for further histological analysis. Cell necrosis was evaluated in 3-μm sections stained with hematoxylin and eosin or DAPI according to the following criteria: cell swelling and lysis, loss of architecture, karyorrhexis, and karyolysis [36]. Plasma levels of carcinoembryonic antigen (CEA) were measured using a human CEA ELISA kit (DRG International, Inc., Springfield, NJ, USA). Plasma levels of aspartate aminotransferase, alanine aminotransferase, and blood urea nitrogen were measured at an outsourcing laboratory (SRL, Inc., Tokyo, Japan).

### 2.25 Statistical analysis

Data are expressed as means ±standard errors of the means (SEM). The sample size was chosen according to previous empirical experience. The experiments were not randomized and the investigators were not blinded to the allocation. Significant differences were statistically analyzed by performing Student's t test, the Tukey–Kramer test, or Dunnett's test as appropriate. A *P* value < 0.05 was considered indicative of a statistically significant difference.

## 3 Results

### 3.1 Acidosis promotes survival of lung cancer cells under low-glucose conditions

**Figure 1:**
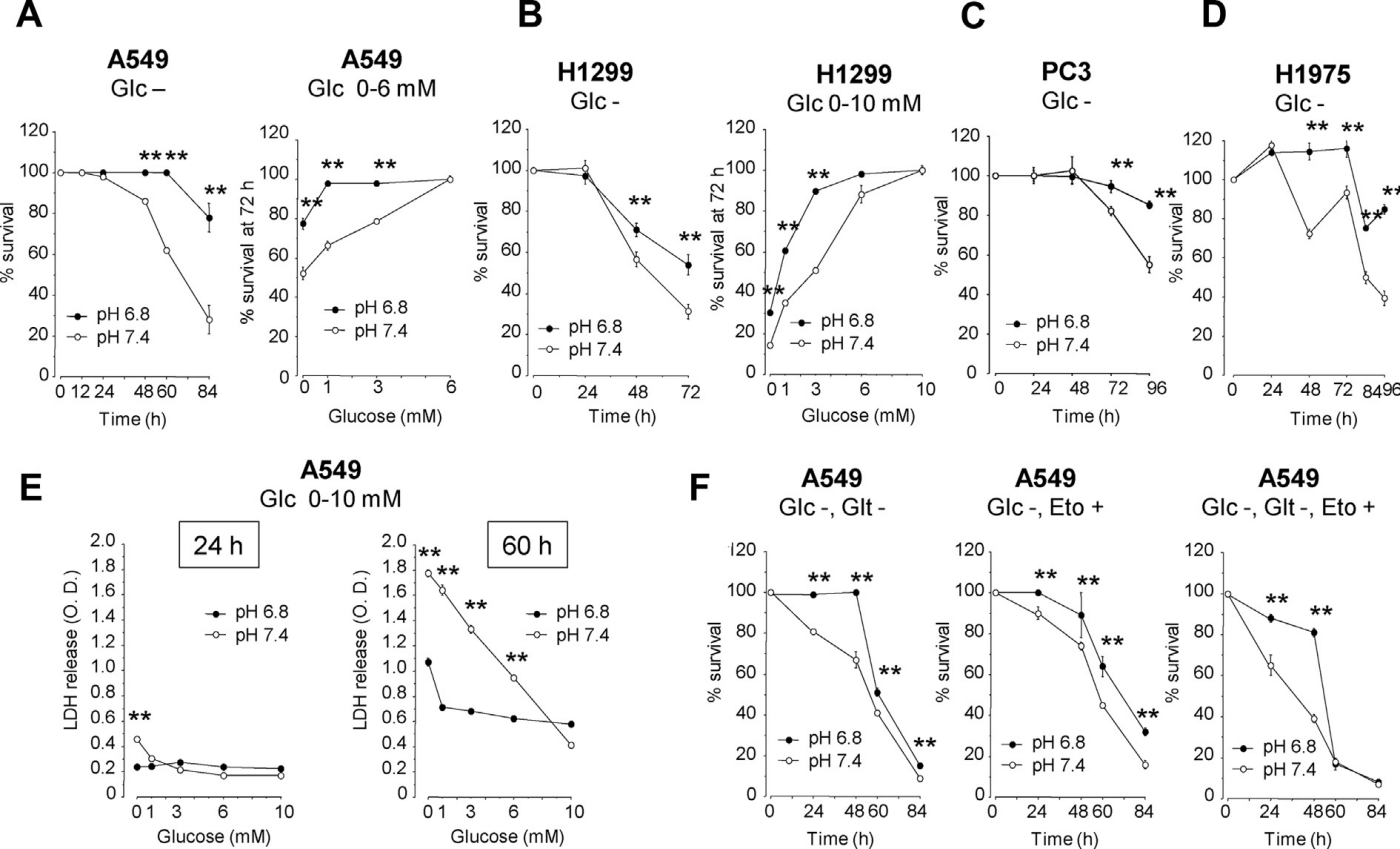
Acidosis promotes the survival of lung cancer cells under low-glucose conditions. A549 (**A**, **E**, and **F**), H1299 (**B**), PC3 (**C**), and H1975 cells (**D**) were grown to confluence, serum-starved, and then incubated for the indicated time periods in medium containing different glucose concentrations in the presence or absence of glutamine or etomoxir, a carnitine palmitoyltransferase1A inhibitor that blocks fatty acid oxidation. Cell survival was assessed by Hoechst 33,342 DNA quantification (**A-B** and **F**) or lactate dehydrogenase (LDH) release assays (**E**). ∗*P* < 0.05 and ∗∗*P* < 0.01 by two-tailed unpaired t tests (n = 6–8). Glc, glucose; Glt, glutamine; Eto, etomoxir.

To examine the effect of acidosis on lung cancer cell survival under low-glucose conditions, the A549, H1299, PC3, and H1975 cells were grown to confluence in growth medium, then serum-starved and incubated in medium at pH 7.4 or 6.8 containing different glucose concentrations. As shown in Figure 1A-E, cell survival decreased with decreasing initial glucose concentrations supplemented in culture medium. However, the cell survival rate in low-glucose medium was significantly higher in acidic (pH 6.8) than in neutral (pH 7.4) medium. The acidosis-induced survival extension in the low-glucose medium was not due to utilization of glutamine or fatty acids because acidosis also promoted cell survival in glucose-free medium lacking glutamine or containing etomoxir, a carnitine palmitoyltransferase-I inhibitor that blocks fatty acid oxidation (Figure 1F). These results indicated that acidosis promoted cell survival under low-glucose conditions.

### 3.2 Acidosis reprograms cancer cell metabolism to save fuel and energy

**Figure 2:**
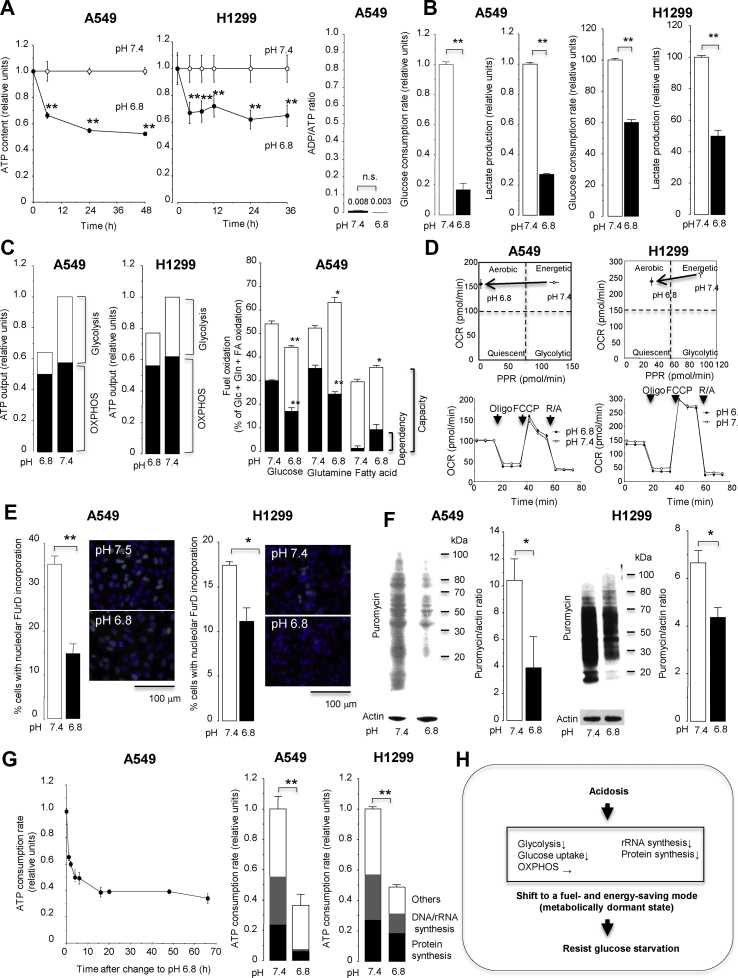
Acidosis reprograms cancer cell metabolism to save fuel and energy. Lung cancer cells were incubated in medium containing 10 mM glucose with pH 6.8 or 7.4 and evaluated for bioenergetic metabolism. (**A**) Intracellular ATP contents and the ADP/ATP ratio. (**B**) The rates of cellular glucose consumption and lactate production. (**C**) A proportional fraction of ATP output from glycolysis and mitochondrial oxidative phosphorylation (OXPHOS) and fuel dependency and capacity in OXPHOS. (**D**) Cellular oxygen consumption rate (OCR) and proton production rate (PPR). Combined measurements of OCR and PPR can provide metabolic phenotypes of cells as aerobic (high OCR and low PPR), energetic (high OCR and high PPR), glycolytic (low OCR and high PPR), or quiescent (low OCR and low PPR). (**E**) Cellular activities of rRNA synthesis determined by in situ nuclear run-on assay and (**F**) protein synthesis determined by surface sensing of translation (SUnSET) assay. (**G**) The total ATP consumption rate and proportional fraction of ATP expenditure. (**F**) Graphical summary of bioenergetic changes induced by acidosis. ∗*P* < 0.05 and ∗∗*P* < 0.01 by two-tailed unpaired t tests (n = 3–8). PPR, proton production rate; FCCP, carbonyl cyanide 4-(trifluoromethoxy)phenylhydrazone; R/A, rotenone and antimycin A; FUrd, fluorouridine.

Acidosis decreased intracellular ATP content without increasing the ADP/ATP ratio, showing a reduced ATP pool while maintaining a proper energy balance (Figure 2A). Acidosis decreased glycolytic ATP production, glucose consumption, and lactate production (Figure 2B,C). Furthermore, acidosis reduced the dependency and capacity of ATP production from mitochondrial oxidative phosphorylation (OXPHOS) with glucose but instead enhanced the capacity of ATP production from OXPHOS with glutamine and fatty acids (Figure 2C), although the basal and maximal activities of OXPHOS were unchanged (Figure 2D). These results indicated that acidosis inhibited glycolysis that consumed a large amount of glucose for fuel but increased relative reliance on more efficient ATP production from OXPHOS using various fuels as energy sources. Acidosis also inhibited the synthesis of rRNA and proteins, representing the major ATP-consuming process [37] (Figure 2E,F) and the cellular import of essential amino acids, reflecting a measure of protein synthesis [38] (Supplementary Fig. S1), and consequently reduced cellular ATP consumption (Figure 2G). Overall, these results indicated that acidosis reprogrammed cellular metabolism to efficiently save glucose (fuel) and ATP (energy) by inhibiting glycolysis and macromolecule synthesis, which enabled lung cancer cells to survive in a metabolically dormant but economic state under low-glucose conditions (Figure 2H).

### 3.3 ESI-09 and HJC0197, previously known inhibitors of EPACs, promote cell death in glucose-free acidic medium independent of EPAC inhibition

**Figure 3:**
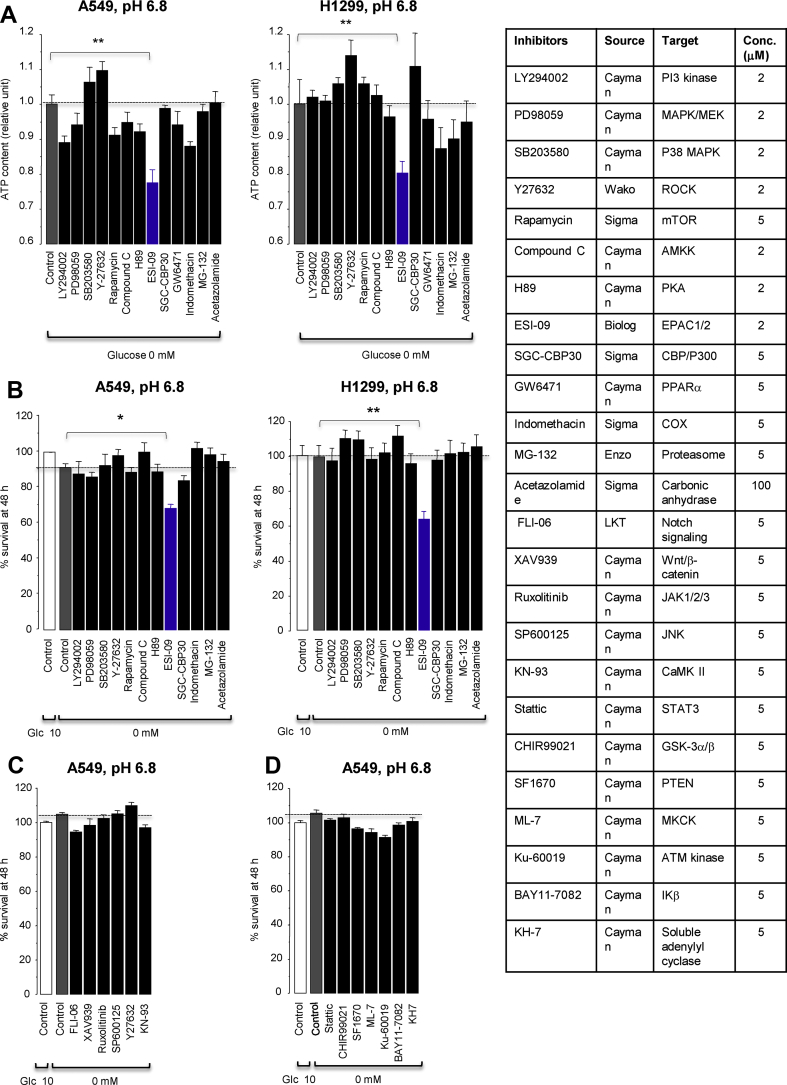
Effects of small-molecule inhibitors on intracellular ATP levels at 24 h (**A**) and survival at 48 h (**B**–**D**) in lung cancer cells cultured in glucose-free acidic medium. ∗*P* < 0.05 and ∗∗*P* < 0.01 by Dunnett's test (n = 8).

**Figure 4:**
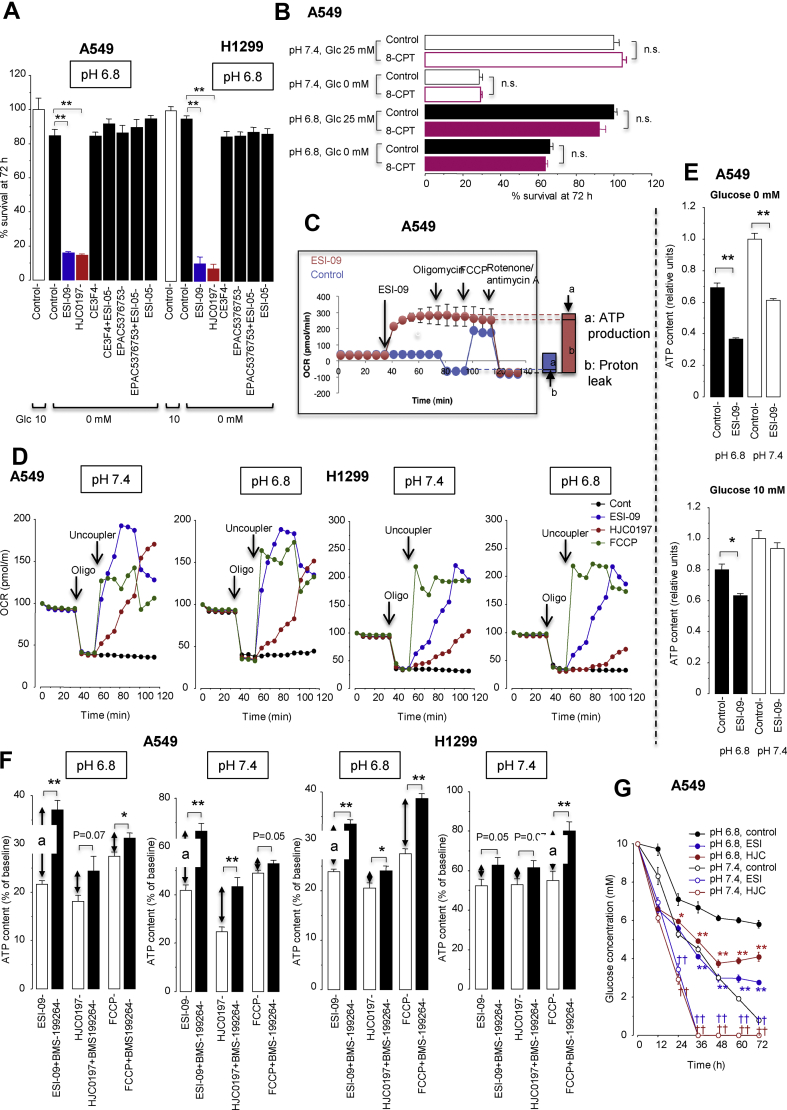
ESI-09 and HJC0197 promote glucose starvation-induced cell death by uncoupling mitochondrial respiration from ATP production independent of EPAC inhibition. (**A**) Effects of various EPAC inhibitors on cell survival under glucose-free conditions at pH 6.8. ∗∗*P* < 0.01 by Dunnett's test (n = 8). (**B**) The EPAC activator 8-pCPT-2′-0-Me-cyclic AMP (8-pCPT) did not improve survival under glucose-free conditions. n.s., not significant by unpaired t tests (n = 8). (**C**) The Seahorse XFp Mito Stress Test profile showing induction of proton leak (mitochondrial uncoupling) by ESI-09 (2 μM) under conditions at pH 7.4. Representative single result of three repetitions (n = 3 in each experiment). OCR, oxygen consumption rate. (**D**) Acidosis did not affect proton leak by ESI-09 (2 μM), HJC0197 (10 μM), and FCCP (2 μM). (**E**) Intracellular ATP contents 18 h after treatment with or without ESI-09 (2 μM) in medium either at pH 7.4 or 6.8 containing or lacking glucose. ∗*P* < 0.05 and ∗∗*P* < 0.01 by two-tailed unpaired t tests (n = 8). (**F**) Increased ATP consumption by F_1_/F_0_ ATP hydrolase contributed to cellular ATP reduction caused by treatment with ESI-09 (2 μM) or HJC0197 (10 μM). *A* indicates the amount of ATP hydrolyzed by the reverse-mode of F_1_/F_0_ ATP synthase as described in the Materials and methods section. ∗*P* < 0.05 and ∗∗*P* < 0.01 by two-tailed unpaired t tests (n = 8). (**G**) Glucose concentrations in culture medium after treatment with ESI-09 (2 μM) or HJC0197 (10 μM). ∗*P* < 0.05 and ∗∗*P* < 0.01 compared with control, pH 6.8, and ^††^*P* < 0.01 compared with control, pH 7.4, by Dunnett's test (n = 8).

Upon screening our in-house small-molecule compounds, we identified ESI-09, a previously known inhibitor of exchange protein directly activated by cAMP (EPAC) 1 and 2, for a relevant hit compound that reduced cellular ATP content and cell survival in glucose-free acidic medium (Figure 3). However, in this medium, no other EPAC inhibitors except for ESI-09 and HJC0197 (Supplementary Fig. S2) inhibited cell survival (Figure 4A), whereas the specific EPAC agonist 8-pCPT-2′-0-Me-cyclic AMP did not support cell survival (Figure 4B). These results indicated that the cytotoxic effects of ESI-09 and HJC-0197 in the glucose-free acidic medium were independent of the inhibition of EPACs.

### 3.4 ESI-09 and HJC0197 promote glucose starvation-induced cell death by uncoupling mitochondrial respiration

The Seahorse Mito stress test assay showed that ESI-09 and HJC0197 induced proton leaks that disengaged oxygen consumption from ATP synthesis (i.e., mitochondrial respiration that was not blocked by the ATP synthase inhibitor oligomycin), indicating that ESI-09 and HJC0197 uncoupled mitochondrial respiration (Figure 4C). Acidosis had no effect on the uncoupling activities of ESI-09, HJC0197, and the classical uncoupler carbonyl cyanide 4-(trifluoromethoxy)phenylhydrazone (FCCP) (Figure 4D). ESI-09 reduced cellular ATP content in the glucose-free medium, either acidic or neutral (Figure 4E), because cells rely on OXPHOS to produce ATP under no-glucose conditions. In contrast, ESI-09 did not reduce cellular ATP content in glucose-rich neutral medium because compensatory glycolysis occurred under that condition (Figure 2B,C). In the glucose-rich and acidic medium, however, ESI-09 moderately reduced cellular ATP content (Figure 4E), probably because acidosis limited glycolysis (Figure 2). In the presence of ESI-09, cells in the glucose-free medium used fatty acids and glutamine to fuel uncoupled mitochondrial respiration (Supplementary Fig. S3).

If mitochondrial respiration is uncoupled (i.e., protons leak through the inner mitochondrial membrane), F_1_F_0_ ATP synthase is presumed to work in reverse mode and hydrolyze ATP to maintain Δψm by pumping protons out of the matrix [39]. Supporting this presumption, the F_1_F_0_ ATP hydrolase inhibitor BMS-199264 [40], which inhibited the maintenance of Δψm in the presence of ESI-09, HJC0197, or FCCP (Supplementary Fig. S4), reduced mitochondrial ATP consumption (Figure 4F). These results showed that ESI-09 and HJC0197 increased ATP dissipation by activating the F_1_F_0_ ATP hydrolase.

ESI-09 and HJC0197 also increased cellular glucose uptake to sustain a compensatory increase in glycolytic ATP production and a futile cycle of OXPHOS (Supplementary Fig. S5). Consequently, ESI-09 and HJC0197 accelerated glucose depletion in the medium, but less severely in the acid than in the neutral medium (Figure 4G) because acidosis limited glucose consumption (Figure 2).

These results indicated that ESI-09 and HJC0197 acted as mitochondrial uncouplers that disengaged fuel oxidation and electron transport from ATP synthesis and consequently not only inhibited ATP production but also created a futile cycle of substrate oxidation and electron transport in an effort to maintain mitochondrial membrane potential, leading to wasting of ATP and fuel (i.e., glucose).

### 3.5 ESI-09 is a potent mitochondrial protonophore

**Figure 5:**
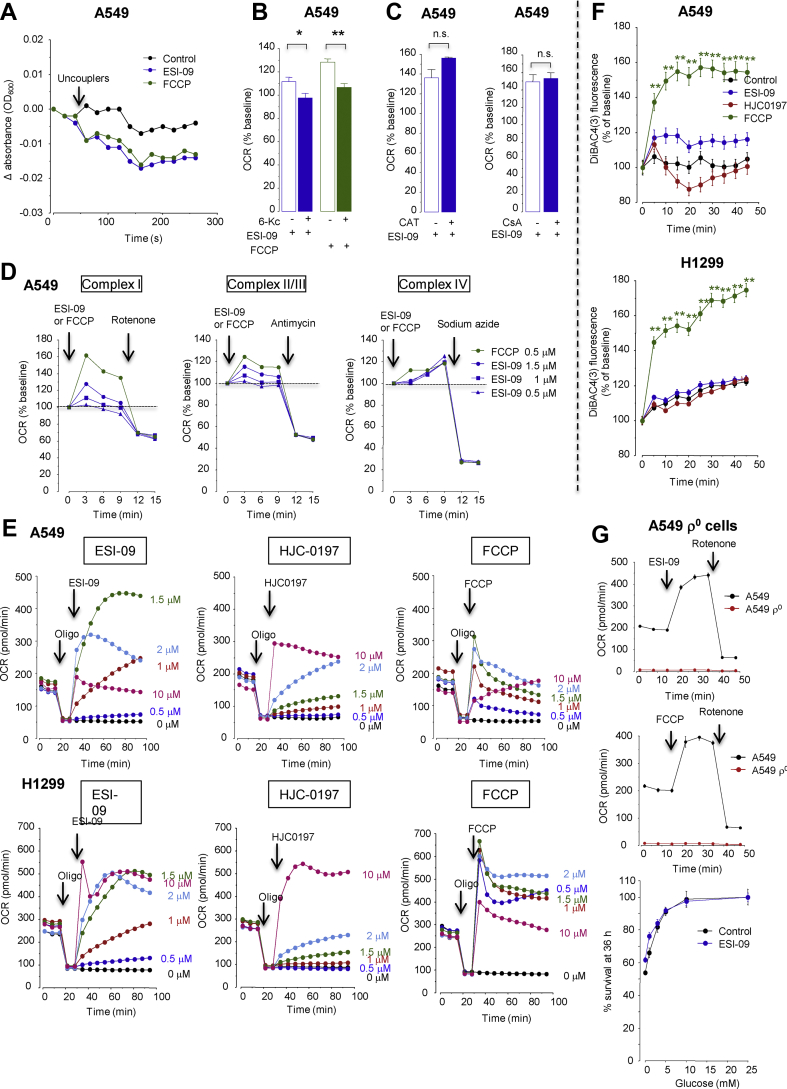
ESI-09 is a potent mitochondrial protonophore. (**A**) ESI-09 induces proton-dependent mitochondrial swelling. Mitochondria isolated from A549 cells were incubated in isotonic acetate buffer in the presence of rotenone and valinomycin and then ESI-09 (1.5 μM) or carbonyl cyanide 4-(trifluoromethoxy)phenylhydrazone (FCCP, 1.5 μM) were added. Absorbance at 600 nm was read out over time. Representative single result of three separate experiments. (**B**) The ESI-09-induced oxygen consumption rate (OCR) increase was dependent on the membrane fluidity. OCR was measured in permeabilized A549 cells respiring on complex 1 substrates in the presence or absence of 6-ketocholestanol (6-Kc, 100 μM), a mitochondrial recoupler that decreases membrane fluidity. ∗*P* < 0.05 and ∗∗*P* < 0.01 by two-tailed unpaired t tests (n = 3). (**C**) ESI-09-induced OCR increase was independent of either adenine nucleotide translocase (ANT) or the permeability transition pore (PTP). OCR was measured in permeabilized A549 cells respiring on complex 1 substrates in the presence or absence of the ANT inhibitor carboxyatractyloside (CAT, 3 μg/ml) or PTP inhibitor cyclosporine A (CsA, 1 μM) (n = 3). n.s., not significant. (**D**) ESI-09 increased OCR in permeabilized A549 cells respiring on complex 1 substrates, complex II/III substrates, and complex IV substrates. Representative single result of three separate experiments. (**E**) Dose-dependent uncoupling effects of ESI-09, HJC0197, and FCCP. Representative single result of three separate experiments. (**F**) Neither ESI-09 nor HJC0197 affected the plasma membrane potential. ∗∗*P* < 0.01 by Dunnett's test (n = 8). (**G**) Effect of ESI-09 on the survival of mitochondria-depleted (ρ^0^) A549 cells (n = 8). A549 ρ^0^ cells were cultured for 36 h in medium at pH 7.4 containing different glucose concentrations in the presence or absence of ESI-09 (1.5 μM) (n = 8). *Upper panels*, changes in OCR after the addition of ESI-09 (1.5 μM) or FCCP (1.5 μM) in A549 cells and A549 ρ^0^ cells (n = 3).

Among the tested EPAC inhibitors, only ESI-09 and HJC0197 showed mitochondrial uncoupling activities (Supplementary Fig. S6), indicating that the induction of mitochondrial uncoupling by ESI-09 and HJC0197 was not due to the inhibition of EPACs. Since ESI-09 and HJC0197 have aromatic structures similar to that of the typical protonohore FCCP, with a similar acid-dissociable group with weak acidic characteristics (pKa: ESI-09, 4.45; HJC-0197, 5.62; and FCCP, 6.2) (Supplementary Fig. S2), we determined if they were bona fide mitochondrial protonophores, especially focusing on ESI-09. Similar to FCCP, ESI-09 induced proton-dependent swelling of isolated mitochondria in potassium acetate buffer containing valinomycin and rotenone [33,34], indicating that ESI-09 transports protons across the mitochondrial inner membrane (Figure 5A). Stimulation of OCR by ESI-09 was abolished in the presence of 6-ketocholestanol (6-Kc), which prevents proton transport through the membrane by decreasing the membrane fluidity [41] (Figure 5B), but not in the presence of cyclosporine A, which inhibits the permeability transition pore (PTP), or carboxyatractyloside, which inhibits the mitochondrial inner membrane adenine nucleotide translocase (ANT) (Figure 5C). ESI-09 increased OCR in permeabilized A549 cells respiring on complex I substrates, complex II/III substrates, and complex IV substrates (Figure 5D). These results indicated that ESI-09 is indeed a protonophore that directly acts on the functional mitochondrial electron transport chain independent of PTP or ANT.

The uncoupling potency of ESI-09 was comparable to that of FCCP; both exhibited uncoupling effects across a concentration range of 1–10 μM, with the maximal activity at 1–2 μM (Figure 5E). However, HJC0197 required a higher concentration (10 μM) to maximize its uncoupling effect (Figure 5E). Furthermore, uncoupling of mitochondria occurred over a longer period (>3 days) with ESI-09 than with the FDA-approved uncoupler niclosamide, which lost its effect within 24 h (Supplementary Fig. S7). Although some uncouplers, such as FCCP, reportedly cause plasma membrane depolarization that may contribute to their non-specific cellular toxicity [42], neither ESI-09 nor HJC0197 affected the plasma membrane potential (Figure 5F). ESI-09 exhibited lower cytotoxicity than FCCP in normal alveolar epithelial cells and normal lung microvascular endothelial cells cultured in normal (glucose-rich, neutral pH) medium (Supplementary Fig. S8). No cytotoxic effects of ESI-09 and FCCP on normal lung fibroblasts were observed in a concentration range <10 μM. ESI-09 did not reduce the survival of mitochondrial DNA-depleted (ρ^0^) A549 cells in the low-glucose medium, which verified that the anti-cancer action of ESI-09 is mediated by mitochondrial deterioration (Figure 5G).

### 3.6 The anti-cancer actions of ESI-09 and HJC0197 depend on glucose concentrations

**Figure 6:**
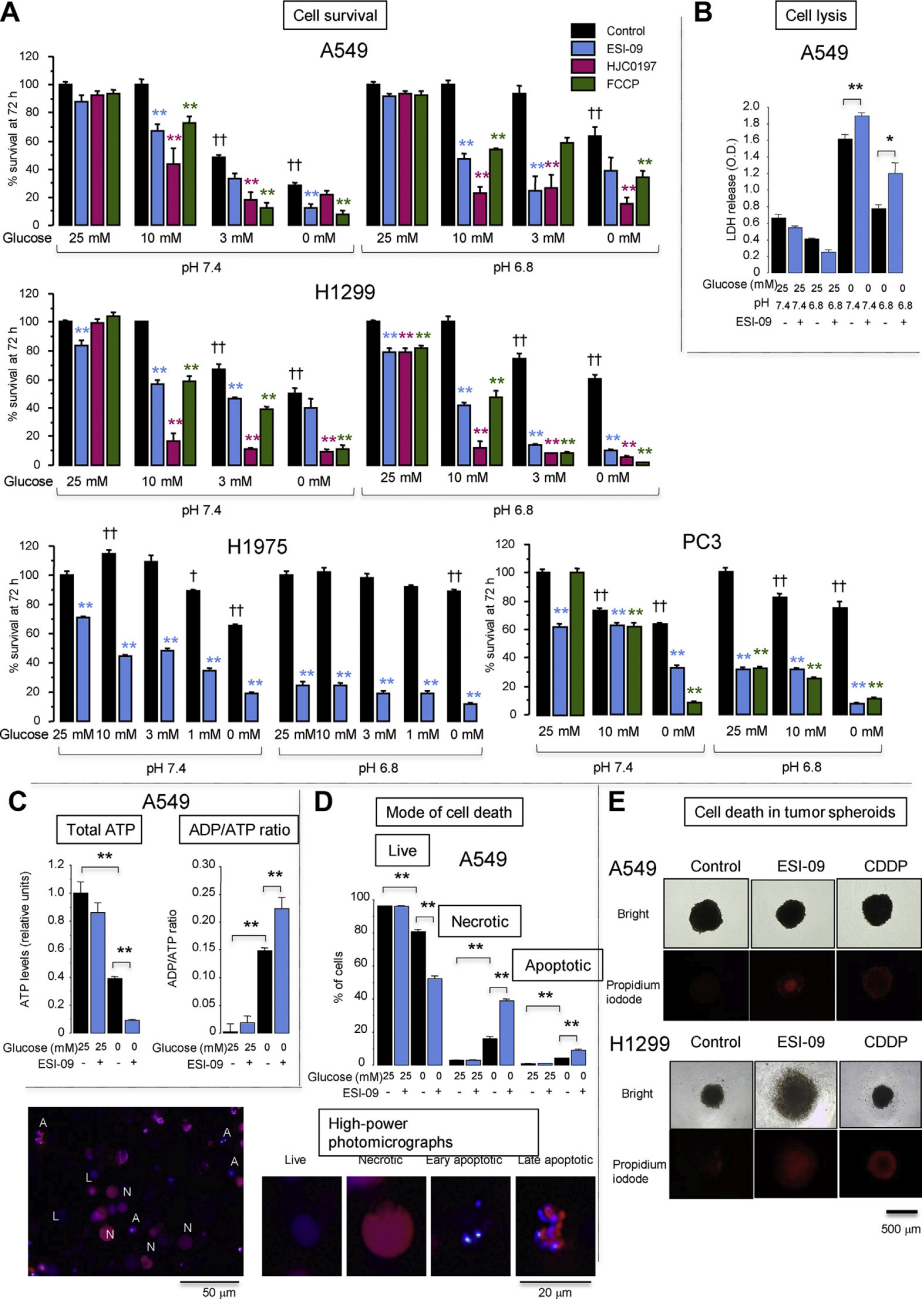
Anti-cancer action of mitochondrial uncouplers depended on the initial concentration of glucose in culture medium. (**A**) Effects of uncouplers on lung cancer cell survival at different initial glucose concentrations. Serum-starved confluent A549, H1299, H1975, and PC3 cells were cultured in medium at pH 7.4 or 6.8 containing different initial glucose concentrations with 4 mM glutamine in the presence or absence of ESI-09 (2 μM), HJC0197 (10 μM), and carbonyl cyanide 4-(trifluoromethoxy)phenylhydrazone (FCCP; 2 μM). Cell survival at 72 h was determined by Hoechst 33,342 DNA quantification assays. ∗∗*P* < 0.01 compared with corresponding controls by Dunnett's test (n = 8). ††*P* < 0.01 and †*P* < 0.05 compared with control at 25 mM glucose at pH 7.4 or 6.8 by Dunnett's test (n = 8). Time course and dose–response effects of the uncouplers on lung cancer cell survival are shown in Supplementary Fig. S9. (**B**) Lactate dehydrogenase (LDH) release from A549 cells cultured for 72 h in medium at pH 7.4 or 6.8 containing or lacking 25 mM glucose in the presence or absence of ESI-09 (2 μM). ∗∗*P* < 0.01 by two-tailed unpaired t tests (n = 4). (**C**) Intracellular ATP content and the ADP/ATP ratio in A549 cells cultured for 48 h in medium at pH 7.4 containing or lacking 25 mM glucose in the presence or absence of ESI-09 (2 μM). ∗∗*P* < 0.01 by Tukey–Kramer tests (n = 8). (**D**) Assessment of the mode of cell death by dual-fluorescence staining with ethidium bromide and Hoechst 33,342. A549 cells were cultured for 48 h in medium at pH 7.4 containing or lacking 25 mM glucose in the presence or absence of ESI-09 (2 μM). ∗∗*P* < 0.01 by Tukey–Kramer tests (n = 4). *L*, live cells; *A*, apoptotic cells; *N*, necrotic cells. (**E**) The 3D spheroids of A549 cells or H1299 cells were treated with ESI-09 (2 μM) or cisplatin (CDDP, 40 μM) for 72 h in medium at pH 7.4 containing 3 mM glucose. Dead cells are visualized in red by propidium iodide staining. CDDP induced cell death more effectively in the periphery rather than central region and vice versa for ESI-09. Representative single result of three separate experiments. (For interpretation of the references to color in this figure legend, the reader is referred to the Web version of this article.)

Our findings of increased glucose demand by cells treated with ESI-09 and HJC0197 (Figure 4G, Supplementary Figs. S5 and S6b) led us to examine whether the anti-cancer actions of these compounds were enhanced when glucose concentrations were reduced, as glucose concentrations in the TME have been shown to be severely reduced [4,12]. When the initial glucose concentration was 25 mM in the neutral pH medium and used to culture cells for 72 h, the survival rate of A549 cells in the presence of ESI-09 (2 μM), HJC0197 (10 μM), or FCCP (2 μM) remained near 100% (Figure 6A). When the initial glucose concentration was reduced to 10 mM, the survival rate of A549 cells in the neutral pH medium remained near 100% in the absence of mitochondrial uncouples, but significantly decreased in the presence of uncouplers. When the initial glucose concentration was reduced to ≤3 mM, the survival rate of A549 cells in the neutral pH medium decreased because of insufficient glucose, but more markedly decreased in the presence of uncouplers compared to their absence. Similarly, the survival rate of the H1299 cells in the neutral pH medium containing 10 mM glucose was nearly 100% in the absence of uncouplers. However, it significantly decreased in the presence of uncouplers, but ESI-09 modestly reduced the survival rates in the neutral medium containing 25 mM glucose (Figure 6A). A similar dependency of uncoupler-induced cell death on reduced initial glucose concentrations was observed in the H1975 and PC3 cells; however, ESI-09 modestly reduced the survival rates in the neutral medium containing 25 mM glucose. Acidosis (pH 6.8) promoted cell survival in the low-glucose medium, reproducing the results displayed in Figure 1. The survival rates of the A549, H1299, H1975, and PC3 cells in the acidic medium containing ≤10 mM glucose were significantly lower in the presence of the uncouplers, although the survival rates of the H1299, H1975, and PC3 cells in the presence of the uncouplers were significantly reduced in the acidic medium containing 25 mM of glucose (Figure 6A). Supplementary Fig. S9a shows the cell survival data at different time points for varying pH and initial glucose concentrations, supporting the enhanced cytotoxic effects of the uncouplers in the low-glucose medium. Supplementary Fig. S9b indicates that the minimum concentration required to significantly reduce cell survival in the glucose-free acidic medium was 0.5–1 μM for ESI-09 in the A549 and H1299 cells, and 2 μM in the H1299 cells and 10 μM in the A549 cells for HJC0197.

In accordance with the cell survival data, ESI-09 induced cell lysis more effectively in the glucose-free medium than in the glucose-rich medium either at pH 7.4 or 6.8 (Figure 6B). In the absence of ESI-09, the A549 cells demonstrated lower intracellular ATP levels with higher ADP/ATP ratios, representing bioenergetic crisis. This crisis was more pronounced in the glucose-free medium than in the glucose-rich medium (Figure 6C). Exposure to ESI-09 decreased the intracellular ATP levels and was associated with an increased ADP/ATP ratio. Again, this was more pronounced in the glucose-free medium than in the glucose-rich medium (Figure 6C). ESI-09 also induced apoptosis and necrosis in the glucose-free medium (Figure 6D). Although varying between cell types, these overall results showed that the anti-cancer action of the uncouplers was enhanced in the low-glucose medium and preserved even in the acidic medium.

The core region in a 3D tumor spheroid can recapitulate several aspects of the TME, including hypoxia, acidosis, low glucose, and chemoresistance [43,44]. Cisplatin induced cell death in tumor spheroids more effectively in the periphery rather than in the core region, which indicated greater chemoresistance of the inner cells (Figure 6E). Conversely, ESI-09 induced cell death in the core rather than peripheral region, showing that ESI-09 killed inner cells that resisted cisplatin. In a 2D monolayer culture, ESI-09 reduced the survival of non-proliferative cells in the glucose serum-starved medium, but not cell proliferation in the glucose serum-rich medium, and vice versa for cisplatin (Supplementary Figs. S10a–S10c). These results showed that ESI-09 effectively killed cancer cells that resisted cisplatin in 2D and 3D culture models. ESI-09 (an EPAC1/2 inhibitor) and ESI-05 (an EPAC2 inhibitor) moderately but significantly inhibited the migration of the A549 cells (Supplementary Fig. S10d).

### 3.7 Diverse effects of mitochondrial uncouplers and electron transport chain (ETC) inhibitors on cancer cell energy metabolism

**Figure 7:**
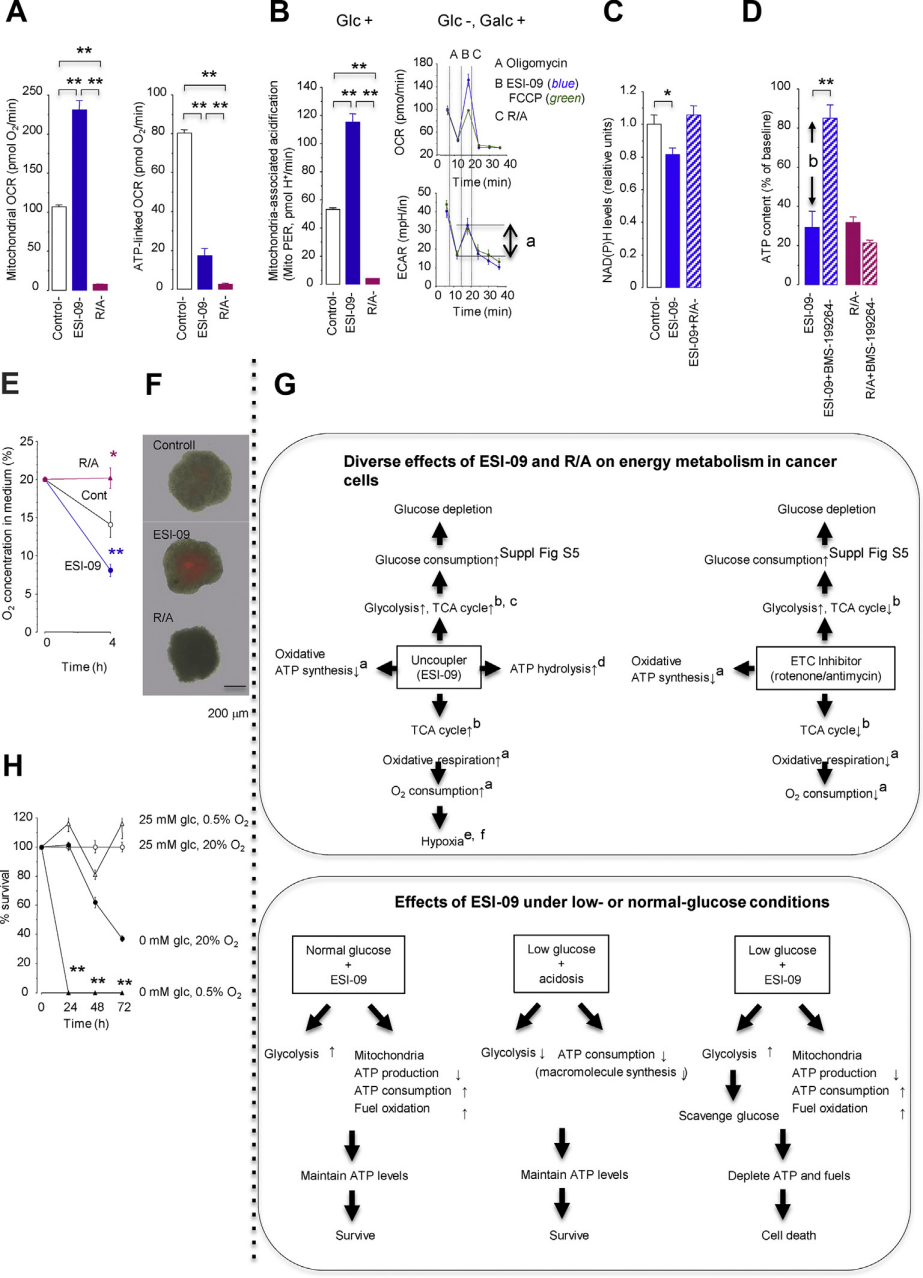
Diverse effects of ESI-09 (2 μM) and the electron transport chain inhibitors rotenone (1 μM) plus antimycin A (1 μM) (R/A) on bioenergetic metabolism in A549 cells. (**A**) Total mitochondrial oxygen consumption rate (OCR; *left*, n = 3) and ATP-linked OCR (*right*, n = 3). (**B**) TCA cycle activity assessed by measuring CO_2_ production in the mitochondrial matrix in the presence (*left*) or absence (but containing galactose, Galc, *right*) of glucose (Glc, n = 3). *A* indicates an increase in mitochondrial CO_2_ production after injection of ESI-09 or carbonyl cyanide 4-(trifluoromethoxy)phenylhydrazone (FCCP). ECAR, extracellular acidification rate. (**C**) Tricarboxylic acid (TCA) cycle activity assessed by the reduction in NAD(P)H, which represents NAD(P)H oxidation (n = 6). (**D**) ATP hydrolysis (n = 8). *B* indicates the amount of ATP hydrolyzed by reverse-mode ATP synthase. (**E**) O_2_ concentration in culture medium from the 2D closed culture system (n = 8). (**F**) Hypoxia in the 3D cell spheroids exposed to ESI-09 or R/A for 40 h. Merged images of bright field and LOX-1 fluorescent pictures (*red*) are shown. (**G**) Graphical summary of the diverse effects of ESI-09 and R/A on cancer cell energy metabolism and the effects of ESI-09 under low- or normal glucose conditions. The superscripts indicate the corresponding figure panels. (**H**) Cell survival in a normoxic (20%) and hypoxic (0.5%) monolayer culture in the presence or absence of glucose (n = 8). (**A**–**F**) ∗*P* < 0.05 and ∗∗*P* < 0.01 by Tukey–Kramer tests. (**H**) ∗∗*P* < 0.01 compared with cells in 0 mM glucose at 20% O_2_ by two-tailed unpaired t tests. (For interpretation of the references to color in this figure legend, the reader is referred to the Web version of this article.)

ETC inhibitors can also target tumor energy metabolism [45]. To optimize the position of mitochondrial uncouplers in cancer therapy, we compared the bioenergetic effects of ESI-09 and the classical ETC inhibitors rotenone (complex I inhibitor) plus antimycin A (complex III inhibitor) (R/A) in lung cancer cells. By inducing a futile cycle of substrate oxidation and electron transport, ESI-09 increased mitochondrial OCR without ATP production (Figure 7A), TCA cycle activity measured by mitochondrial CO_2_ production (Figure 7B) and reduction of cellular NAD(P)H levels (Figure 7C), and glucose demand for increased glycolysis and non-glycolysis (i.e., the TCA cycle, etc.) (Supplementary Fig. S5). In contrast, the ETC inhibitors R/A decreased mitochondrial OCR (Figure 7A), TCA cycle activity (Figure 7B), and glucose demand for non-glycolysis but increased glucose demand for increased glycolysis (Supplementary Fig. S5). Furthermore, ESI-09, but not R/A, activated F_1_F_0_ ATP hydrolase and mitochondrial ATP consumption (Figure 7D). ESI-09, but not R/A, also reduced oxygen levels in 2D closed-monolayer cells (Figure 7E) and enhanced hypoxia in the central region of 3D spheroids (Figure 7F).

In summary, although both ESI-09 and R/A inhibited mitochondrial ATP production and enhanced glycolysis and glucose consumption, only ESI-09, but not R/A, enhanced TCA cycle activity, OCR (without ATP synthesis), mitochondrial ATP consumption, and hypoxia (Figure 7G). Increased glucose consumption associated with oxygen deprivation by ESI-09 is probably important because theoretically, cancer cells no longer survive when both glucose and oxygen are scarce (Figure 7H).

### 3.8 Anti-tumor activity of ESI-09 in A549 cell xenograft mice

**Figure 8:**
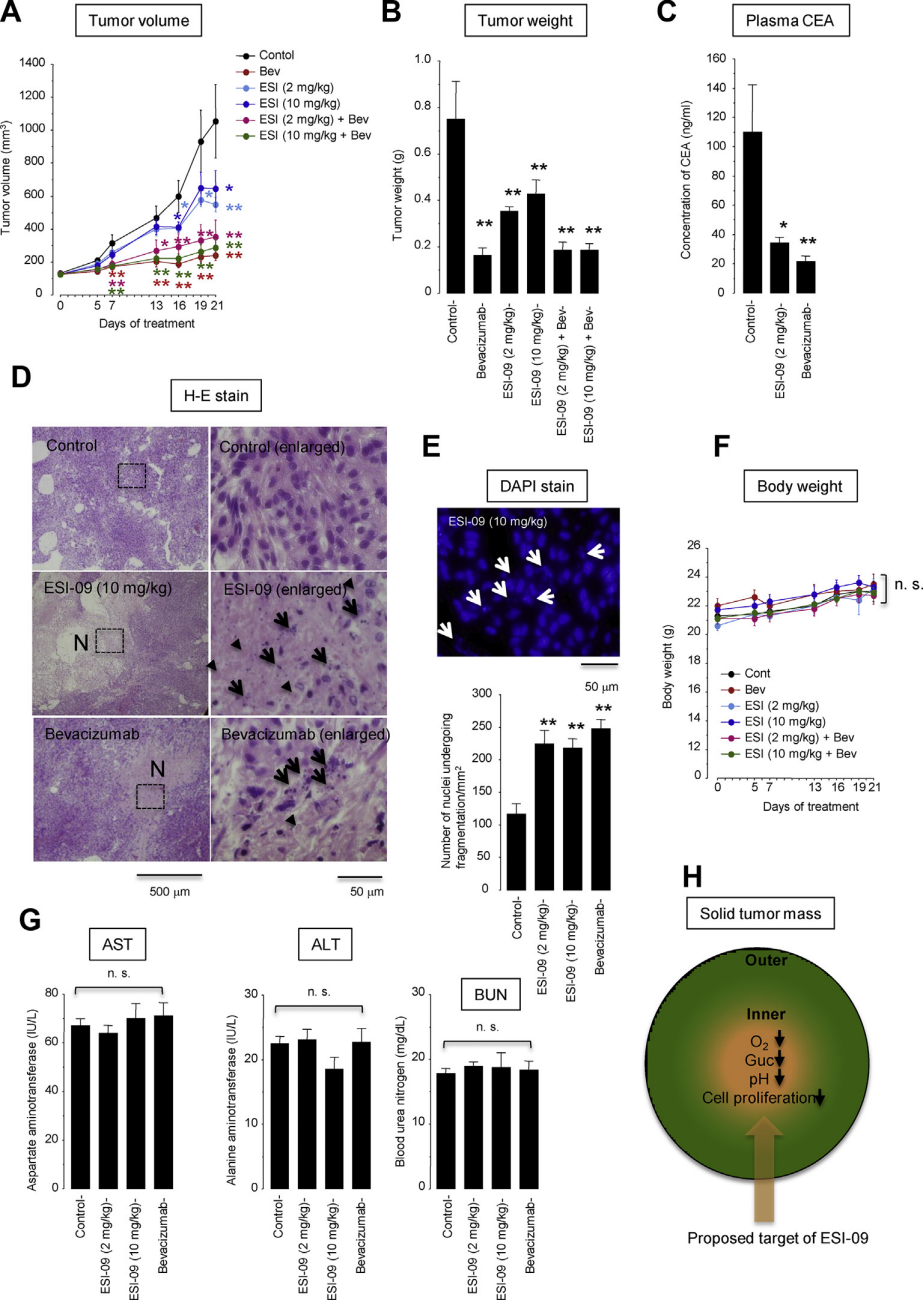
Effects of ESI-09 and bevacizumab on lung cancer xenograft models with A549 cells. Mice were treated with vehicle (n = 6), ESI-09 (2 mg/kg, daily, i.p.) (n = 7), ESI-09 (10 mg/kg, daily, i.p.) (n = 7), bevacizumab (Bev, 5 mg/kg, twice weekly, i.p., n = 7), Bev (5 mg/kg, twice weekly, i.p.) plus ESI-09 (2 mg/kg, daily, i.p.) (n = 7), or Bev (5 mg/kg, twice weekly, i.p.) plus ESI-09 (10 mg/kg, daily, i.p.) (n = 7). We removed from analysis one animal in the ESI-09 (2 mg/kg) group because of accidental death on day 2 and one animal in the control group because no tumor growth was observed over the experimental time period. Data are shown after removing these animals. ∗*P* < 0.01 and ∗*P* < 0.05 compared with the control group by Dunnett's test. n.s., not significant. (**A**) Tumor volume over time. Comparisons were made with the control group on each day of treatment. (**B**) Tumor weight on day 21. (**C**) Plasma carcinoembryonic antigen (CEA) concentration. One animal in the ESI-09 (2 mg/kg) group was not tested because of a sample volume shortage. (**D**) Hematoxylin-eosin staining of paraffin-embedded tissue sections from xenografted tumors. The right panels expand the area boxed by the dashed lines. *N* denotes a pale pink area consisting of necrotic cells with nuclear fragmentation (karyorrhexis, *arrows*) and fading (karyolysis, *arrowheads*). (**E**) Number of 4′,6′-diamidino-2-phenylindole (DAPI)-stained nuclei undergoing nuclear fragmentation (karyorhexis, *arrows*). (**F**) Body weight. (**G**) Plasma levels of aspartate aminotransferase (AST), alanine aminotransferase (ALT), and blood urea nitrogen (BUN). (**H**) Proposed target of ESI-09 inside tumor mass. Glc, glucose. (For interpretation of the references to color in this figure legend, the reader is referred to the Web version of this article.)

The in vivo anti-tumor activity of ESI-09 was evaluated in A549-cell xenograft mice. Because impeding angiogenesis would promote glucose depletion and thus might improve the efficacy of ESI-09, we tested the effects of a monotherapy with ESI-09 (2 mg/kg or 10 mg/kg, daily, i.p.), a monotherapy with the anti-vascular endothelial growth factor antibody bevacizumab (5 mg/kg, twice weekly, i.p.), and a combination therapy with ESI-09 plus bevacizumab (Figure 8). The monotherapy with ESI-09 at 2 mg/kg or 10 mg/kg significantly inhibited tumor growth by day 16 and beyond while the monotherapy with bevacizumab inhibited tumor growth more rapidly, as early as day 7 and beyond (Figure 8A). The monotherapy with ESI-09 or bevacizumab also reduced the tumor volume on the day of sacrifice (day 21) (Figure 8B). Because bevacizumab almost completely arrested tumor growth, we could not detect any additive effect of ESI-09 to bevacizumab on tumor growth. The plasma CEA levels were also significantly decreased in the mice treated with either ESI-09 or bevacizumab alone (Figure 8C). Tissue sections of tumors obtained from the mice treated with ESI-09 or bevacizumab showed an abundance of necrotic lesions containing dying cells undergoing karyorrhexis and karyolysis, which indicated oncotic necrosis [36] (Figure 8D,E). Both ESI-09 and bevacizumab were well tolerated with no significant effects on body weight (Figure 8F) or AST, ALT, and BUN plasma levels (Figure 8G). These results showed that treatment with ESI-09 caused tumor cell necrosis and inhibited tumor growth in vivo.

## 4 Discussion

We reported two major findings in this study. First, we demonstrated that acidosis limited cellular consumption of glucose and ATP, which caused tumor cells to enter a metabolically dormant but energetically economic state that supported tumor cell survival during glucose starvation. Second, we identified two known EAPC inhibitors, ESI-09 and HJC0197, as compounds that effectively killed tumor cells in the low-glucose medium at either acidic or neutral pH. ESI-09 (and also HJC0197, albeit less potent than ESI-09) acted as a mitochondrial uncoupler independent of EPAC inhibition that disengaged fuel oxidation and electron transport from ATP synthesis; as a result, it not only inhibited ATP production but also created a futile cycle of substrate oxidation and electron transport in an effort to maintain mitochondrial membrane potential, leading to wasting of ATP and fuel (i.e., glucose). Less cytotoxicity of ESI-09 under normal-thlower glucose conditions will be ideal for therapeutic translation in reducing side effects in normal healthy tissue and selectively killing tumor cells in a low-glucose microenvironment. We conclude that ESI-09 is a potent anti-cancer uncoupler that can target metabolic vulnerability to low-glucose conditions, which are often acidic (Figure 8H).

We found that the acidosis-induced metabolic reprogramming to save energy (ATP) and fuel (glucose) involved two distinct mechanisms of action by acidosis: first, the inhibition of glycolysis that consumed a large amount of glucose as fuel and second, the inhibition of rRNA/protein synthesis that consumed a large amount of ATP. Our findings are supported by those of several previous studies. For example, acidosis has been shown to inhibit pH-sensitive glycolytic enzyme phosphofructokinase [46] and stimulate the MondoA/thioredoxin-interacting protein (TXNIP) pathway that inhibits the glycolytic phenotype [20,22]. Acidosis has also been shown to suppress rRNA/protein synthesis by inhibiting the mammalian target of rapamycin (mTOR) either via the stimulation of AMP-activated protein kinase [20] or the acid-mediated dispersion of lysosomes [23] by disconnecting ribose synthesis from the pentose phosphate pathway [21] and detaining von Hippel-Lindau tumor suppressor protein with the promoter of rRNA genes [19]. These lines of evidence together with our own validate the presented concept that acidosis reduces the overall metabolic rate, rendering tumor cells tolerant to glucose starvation stress. On the basis of this concept, we speculate that tumor cells gage their environmental pH and if it becomes low, they limit the metabolic rate to prepare for impending glucose shortage due to increased glycolytic metabolism and poor perfusion in the TME.

Although many chemotherapeutic agents require active cell proliferation to be effective, ESI-09 and HJC0197 can target non-proliferative, metabolically restricted, quiescent cells, which have been shown to often resist chemotherapy [47]. ESI-09 and HJC0197 induced energetic crises in tumor cells under low-glucose stress, even associated with acidosis, by three combined mechanisms: decreasing ATP production due to uncoupled respiration from ATP synthesis, increasing ATP dissipation via the reversal action of the F_0_F_1_ ATPase to maintain the mitochondrial membrane potential, and scavenging available fuels (glucose and O_2_) by a futile cycle of substrate oxidation. Using the analogy of energy production from an automobile engine, we propose that acidosis probably shifts engine operation to an economical mode with lower power (ATP) output but less fuel (glucose and O_2_) consumption. Unlike ETC inhibitors, which just turn off the engine (mitochondrial respiration), mitochondrial uncouplers, such as ESI-09 and HJC0197, are just as likely to rev the engine by keeping the gas pedal in neutral, which continues wasteful consumption of fuels (glucose and O_2_) and energy (ATP) to maintain futile engine rotation without any substantial energy generation (that is, mitochondrial respiration decoupled from ATP synthesis in association with increased ATP consumption). There is a natural scarcity of nutrients and O_2_ in the TME. If mitochondrial uncouplers force tumor cells to scavenge all available nutrients, such as glucose, glutamine, and fatty acids, or at least both glucose and O_2_, they would no longer survive (Figure 7H). Theoretically, energy depletion and fuel exhaustion caused by mitochondrial uncoupling are universal features not associated with resistance to therapy, unlike targeting a specific oncogenic pathway that is mutated in certain tumors or a specific metabolic pathway that is flexible and redundant [48].

In a mouse xenograft model with A549 cells, significant inhibition of tumor growth was observed on day 16 and beyond after treatment with ESI-09 but as early as day 7 and beyond after treatment with bevacizumab (Figure 8A). The difference in onsets of the anti-tumor effects of ESI-09 vs bevacizumab may be due to their different mechanisms of action; our in vitro data from the A549 cells showed that the anti-tumor action of ESI-09 required a reduced environmental glucose concentration, which could not be achieved until the tumor size substantially increased; in contrast, bevacizumab inhibited tumor growth from an early stage of treatment by impeding angiogenesis. Unfortunately, we could not observe any add-on effects of ESI-09 to bevacizumab because treatment with bevacizumab alone almost completely arrested tumor growth in our experiments.

ESI-09 and HJC0197 have been identified in high-throughput screening as specific competitive inhibitors that target the cAMP-binding domain of EPAC/cAMP-guanine exchange factor (GEF) [[49], [50], [51], [52], [53], [54]] (Supplementary Fig. S2). However, we showed that mitochondrial uncoupling by ESI-09 and HJC0197 was not due to their inhibitory effects on EPACs, but rather by the lipophilicity of their structural formulas that enabled passage through the mitochondrial inner membrane and their weak acidity (predicted p*K*a of ESI-09 = 4.45 and predicted p*K*a of HJC0197 = 5.62) that enabled partial and reversible pH-dependent polarization. ESI-09 has several advantages over previous uncouplers. ESI-09 had an effective concentration range of 1–10 μM, which was very close to that of the classical uncoupler FCCP, but did not depolarize the plasma membrane, in contrast to FCCP (Figure 5F), and exhibited less cytotoxicity to non-cancerous cells under normal culture conditions (Supplementary Fig. S8). Furthermore, the uncoupling effect of ESI-09 was more durable than that of niclosamide, an FDA-approved anthelminthic uncoupler (Supplementary Fig. S7).

However, the clinical application of ESI-09 for cancer therapy may impose several challenges. First, mitochondrial uncouplers generally have a narrow therapeutic window between efficacy and toxicity [55]. For example, the historic uncoupler 2,4-dinitrophenol (DNP) used for weight loss in the 1930s was banned because of serious damage to the liver, skeletal muscle, heart, and eyes [56]. Niclosamide is currently used as an oral uncoupler effective for tapeworm, although its low oral bioavailability explains its good tolerability and limits its applications in cancer therapy [57,58]. Although the safety and bioavailability of ESI-09 have not been tested in humans, several animal studies, including the present work, have shown it to be safe and well-tolerated in vivo. In fact, mice treated with ESI-09 as an EPAC inhibitor at a range of 2 (our own study) to 50 mg/kg/day [59] over a ≤ 3-week period showed no noticeable toxicity [[59], [60], [61], [62], [63]]. Second, the weak acidity of ESI-09 may affect the functions of non-mitochondrial organelles that have lower pH [55], although conversely, it may be helpful for reaching a high intracellular concentration of ESI-09 in an acidic TME [64]. Third, treatment with mitochondrial uncouplers may aggravate vascular occlusive diseases because it may reduce nutrients and O_2_ not only in tumors but also in non-tumorous tissue in poorly vascularized organs. Conversely, however, uncouplers have been reported to prevent ischemia-reperfusion injury by reducing ROS production [65]. Fourth, it is also possible that the selective targeting of uncouplers to glucose-deficient cells would reduce unwanted damage to normal, healthy, well-nourished cells but may also reduce a desirable efficacy on well-nourished actively proliferating tumor cells. Thus, a combination therapy with ESI-09, which targets poorly nourished non-proliferative tumor cells, and chemoradiation, which targets well-nourished proliferating tumor cells, may be preferable, as our data showed that cisplatin effectively killed cancer cells resistant to ESI-09 and vice versa (Figure 6E and Supplementary Fig. S10). However, the concurrent use of uncouplers might decrease sensitivity to chemoradiation by promoting tumor hypoxia (Figure 7F) [66].

ESI-09 might exert its anti-cancer action not only by mitochondrial uncoupling but also by EPAC inhibition because EPACs, which catalyze the exchange of guanosine 5′-diphosphate and guanosine 5′-triphosphate (GTP) on the small GTPase Rap1 and Rap2, are actively involved in cell adhesion and migration [67]. Recent studies have shown that the inhibition of EPACs suppresses migration and metastasis in various cancer cell lines [50,[68], [69], [70], [71], [72], [73], [74], [75]], consistent with our findings of a moderate but significant inhibition of A549 cell migration by ESI-09 (Supplementary Fig. S10d). It was also possible that ESI-09 might exert its anti-cancer action through extramitochondrial deterioration since some mitochondrial uncouplers have been shown to inhibit several oncogenic pathways, such as Wnt/β-catenin, mTORC1, STAT3, and Notch [57,58]. However, this possibility was unlikely in our case, because in our study, none of the specific inhibitors for these signaling molecules inhibited tumor cell survival in the glucose-free acidic medium, failing to reproduce the anti-cancer action of ESI-09 (Figure 3).

In conclusion, we identified ESI-09 as a potent uncoupler that can target metabolically vulnerable tumor cells to low-glucose stress even under acidic conditions. The higher cytotoxicity under lower-than-normal glucose conditions suggests that ESI-09 could be safer than conventional chemotherapy for reducing side effects on normal tissue, targeting metabolic dormancy, treating a variety of cancer cell types unlike many drugs specifically targeting genomic alterations, and combating tumor metabolic flexibility and redundancy [48]. Thus, ESI-09 potentially can be used to overcome resistance to current cancer therapies.

## Funding

This study was supported by a grant-in-aid for scientific research from the Ministry of Education, Science, and Culture, Japan (grant number: 19K17685; recipient: Y.M.).

## Author contributions

Conceptualization, K.A. Methodology, Y.M., R.K., T.T., and K.A. Investigation, Y.M., R.K., J.K., and K.A. Validation, N.K., K.Y., and H.N. Writing original draft, Y.M. and K.A. Review and editing, K.A., N.K., and H.N.
