# Supplementary material for: Anti-cancer strategy targeting the energy metabolism of tumor cells surviving a low-nutrient acidic microenvironment
Source: Mol Metab. 2020 Sep 30;42:101093. doi: 10.1016/j.molmet.2020.101093 (PMC7578269; doi:10.1016/j.molmet.2020.101093)
Supplement: Multimedia component 1 [file mmc1.docx]

**Supplementary Information**

**Anticancer strategy targeting the energy metabolism of tumor cells surviving a low-nutrient acidic microenvironment**

**Yuki Maeda, Ryota Kikuchi, Junichiro Kawagoe, Takao Tsuji, Nobuyuki Koyama, Kazuhiro Yamaguchi, Hiroyuki Nakamura, Kazutetsu Aoshiba**

**SUPPLEMNTARY TABLE AND FIGURES**

**Table S1**. Reagents used in this study.

| Reagents | Source, catalog # | Solvent | Final conc. |
| --- | --- | --- | --- |
| ESI-09 | Biolog, B133 | DMSO | 0.5*–*10 μM (in vitro)  2 or 10 mg/kg (in vivo) |
| HJC0197 | Cayman, 19092 | DMSO | 0.5*–*10 μM |
| CE3F4 | Cayman, 17767 | DMSO | 10 μM |
| (R)-CE3F4 | Tocris, 4793 | DMSO | 100 μM |
| ESI-05 | Biolog, M092 | DMSO | 10 μM |
| EPAC5376753 | Focus Biom., 10-1564 | DMSO | 10 μM |
| GGTI 298 | Cayman, 16176 | DMSO | 10 μM |
| 8-pCPT-2′-O-Me-cAMP | Cayman, 17143 | DMSO | 0.1 μM |
| FCCP | Cayman, 15218 | DMSO | 0.5*–*10 μM |
| Niclosamide | Adipogen, AG-CR1-3644 | DMSO | 0.5*–*10 μM |
| 6-ketocholestanol | SantaCruz, 210630 | EtOH | 100 μM |
| Cyclosporine A | TCI, C2408 | DMSO | 1 μM |
| Carboxyatractyloside | Cayman, 21120 | DMSO | 3 μg/ml |
| Oligomycin A | Cayman, 11342 | DMSO | 0.5*–*2 μM |
| Rotenone | Cayman, 13995 | DMSO | 0.5*–*2 μM |
| Antimycin A | Enzo, ALX-380-075 | DMSO | 0.5*–*2 μM |
| Etomoxir | Cayman, 11969 | DMSO |  |
| BMS-199264 | Sigma, BM0017 | DMSO | 0.5–2 μM |
| Cycloheximide | Sigma, 01810 | DW | 2.5 mM |
| Actinomycin D | Sigma, A9415 | DMSO | 8 μM |
| Cisplatin | Nippon Kayaku | - | 2*–*40 μM |
| Bevacizumab | Chugai Pharmaceutical | - | 5 mg/kg (in vivo) |
| LY294002 | Cayman, 70920 | DMSO | 2 μM |
| PD98059 | Cayman, 10006726 | DMSO | 2 μM |
| SB203580 | Cayman, 13067 | DMSO | 2 μM |
| Y27632 | Wako, 030-24026 | DMSO | 2 μM |
| Rapamycin | Sigma, R0395 | DMSO | 5 μM |
| Compound C | Cayman, 11967 | DMSO | 2 μM |
| H89 | Cayman, 10010556 | DMSO | 2 μM |
| SGC-CBP30 | Sigma, SML1133 | DMSO | 5 μM |
| GW6471 | Cayman, 11697 | DMSO | 5 μM |
| Indomethacin | Sigma, 18280 | DMSO | 5 μM |
| MG-132 | Enzo, BML-PI102 | DMSO | 5 μM |
| Acetazolamide | Sigma, A6011 | DMSO | 100 μM |
| FLI-06 | LKT, F4432 | DMSO | 5 μM |
| XAV939 | Cayman, 13596 | DMSO | 5 μM |
| Ruxolitinib | Cayman, 11609 | DMSO | 5 μM |
| SP600125 | Cayman, 10010466 | DMSO | 5 μM |
| KN-93 | Cayman, 13319 | DMSO | 5 μM |
| Stattic | Cayman, 14590 | DMSO | 5 μM |
| CHIR99021 | Cayman, 13122 | DMSO | 5 μM |
| SF1670 | Cayman, 15368 | DMSO | 5 μM |
| ML-7 | Cayman, 11801 | DMSO | 5 μM |
| Ku-60019 | Cayman, 17502 | DMSO | 5 μM |
| BAY11-7082 | Cayman, 10010266 | DMSO | 5 μM |
| KH-7 | Cayman, 13243 | DMSO | 5 μM |
| 5-Fluorouridine | TCI, F0636 | DW | 2 μM |
| Puromycin | Nakarai, 29455 | DMSO | 2 μM |

Abbreviations: DMSO, dimethyl sulfoxide; DW, distilled water; EtOH, ethanol

**Supplementary Figure 1**

**
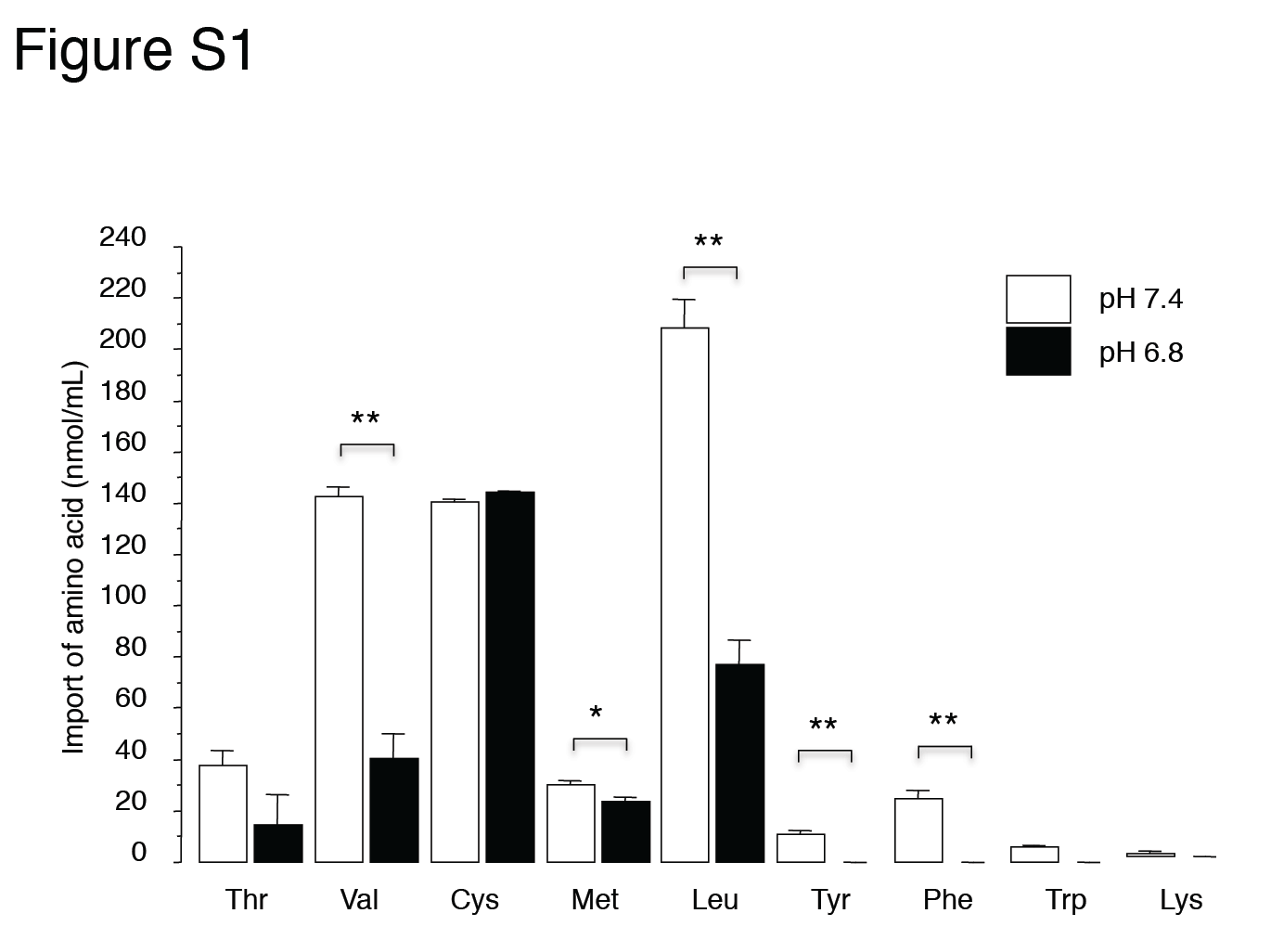
**

**Fig. S1.** Acidosis inhibits the cellular import of essential amino acids, which represents a proportional measure of protein synthesis. Serum-starved confluent A549 cells were cultured for 40 h in acidic (pH 6.8) or neutral pH (7.4) medium containing 25 mM glucose, and the concentrations of essential amino acids in medium were measured by liquid chromatography–mass spectrometry. **P* < 0.05, ***P* < 0.01 by two-tailed unpaired *t* test (n = 4).

**Supplementary Figure 2**


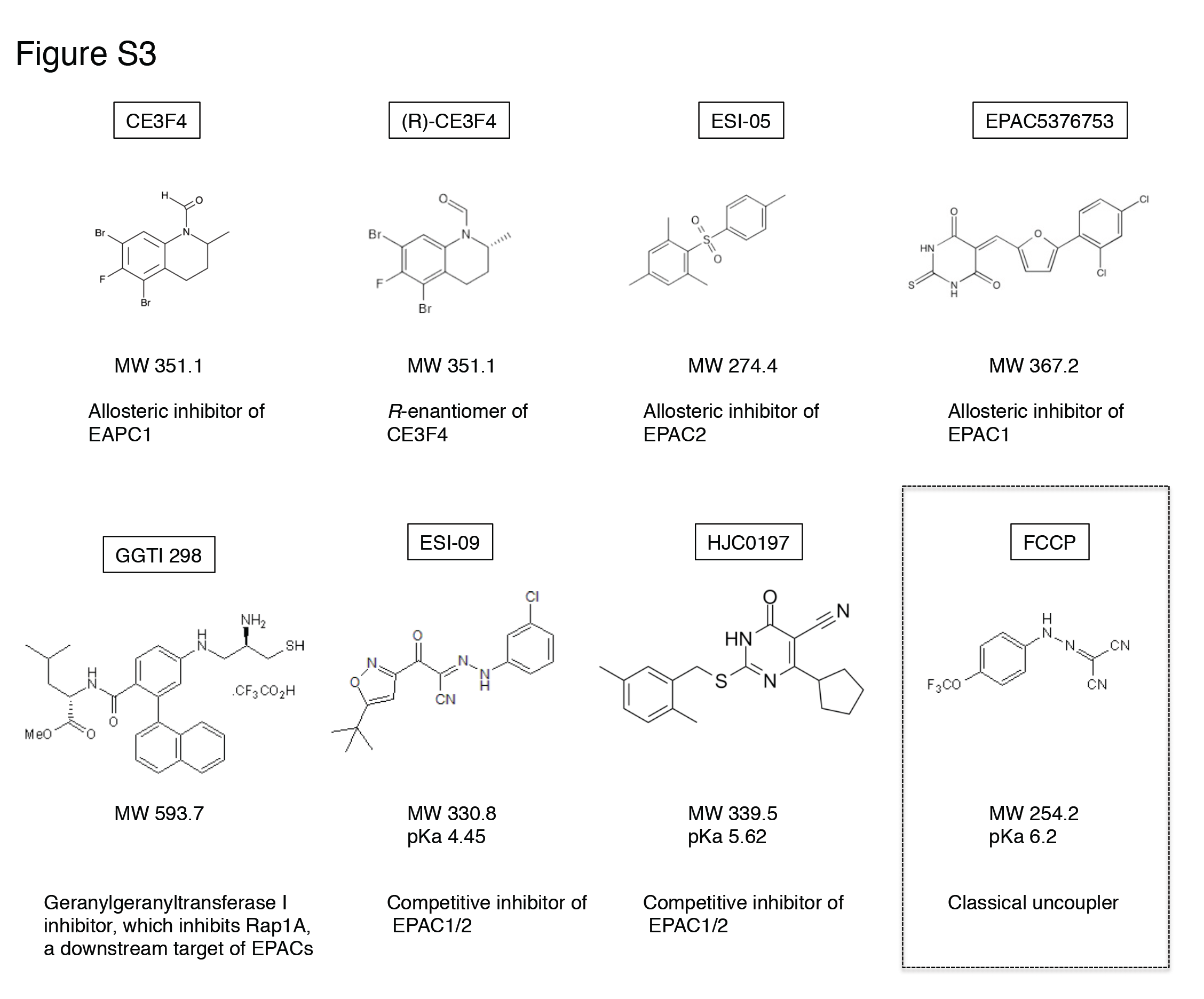


**Fig. S2.** Structural formulas of EPAC inhibitors and the classical mitochondrial uncoupler FCCP.

**CE3F4:** 5,7-dibromo-6-fluoro-3,4-dihydro-2-methyl-1(2H)-quinolinecarboxaldehyde (CE3F4) is a specific allosteric EPAC1 inhibitor that blocks EPAC1-induced Rap1 activation (IC_50_ = 23 μM) [1].

**(R)-CE3F4**: R-enantiomer of CE3F4 more potent than CE3F4 (IC_50_ = 4.2 and 44 μM for EPAC1 and EPAC2, respectively) [1].

**ESI-05:** 1,3,5-Trimethyl-2-[(4-methylphenyl)sulfonyl]benzene (ESI-05) is a specific competitive EPAC2 antagonist of EPAC2-mediated GEF activity (IC_50_ = 0.4 μM) [2].

**EPAC5376753:** (5-((5-(2,4-Dichlorophenyl)furan-2-yl)methylene)-2-thioxodihydropyrimidine-4,6(1H,5H)-dione) is an allosteric inhibitor of EPAC1, IC_50_= 4 μM [3].

**GGTI 298:** a geranylgeranyltransferase I inhibitor that inhibits Rap1A, a downstream target of EPACs.

**ESI-09 and HJC0197:** 3-(5-tert-Butyl-isoxazol-3-yl)-2-[(3-chlorophenyl)-hydrazono]-3-oxo-propionitrile (ESI-09) and 4-cyclopentyl-2-[[(2, 5-dimethylphenyl)methyl]thio]-1, 6-dihydro-6-oxo-5-pyrimidinecarbonitrile (HJC0197) belong to a group of small-molecule compounds that were identified as specific competitive antagonists for EPAC1 and EPAC 2 during high-throughput screening [4-9]. Previous studies confirmed that ESI-09 and HJC0197 bind to the cAMP-binding domain of EPAC/cAMP-guanine exchange factor (GEF) and selectively inhibit cAMP-dependent EPAC GEF activity (ESI-09: IC_50_ = 1.4 and 3.2 µM, respectively, against EPAC2 and EPAC1; [cAMP] = 25 µM) [5, 9]; HJC0197: IC_50_ = 5.9 µM for EPAC2) [4, 6]. They have no effect on cAMP-induced type I and II protein kinase A activity. In addition, ESI-09 has no significant inhibitory effect on phosphodiesterases but does have very weak inhibitory activities toward hERG and CYP450 enzymes [4]. When used at high concentrations >50 μM that were much higher than the effective pharmacological concentrations (<10 μM), they exhibit nonspecific effects on protein stability [10].

**FCCP:** Carbonyl cyanide 4-(trifluoromethoxy)phenylhydrazone (FCCP) is a classical mitochondrial uncoupler.

**References**

1. Courilleau D, Bouyssou P, Fischmeister R, Lezoualc'h F, Blondeau JP. The (R)-enantiomer of CE3F4 is a preferential inhibitor of human exchange protein directly activated by cyclic AMP isoform 1 (Epac1). Biochem Biophys Res Commun. 2013;440*:*443. doi: 10.1016/j.bbrc.2013.09.107.
2. Tsalkova T, Mei FC, Li S, Chepurny OG, Leech CA, Liu T, Holz GG, Woods VL Jr, Cheng X. Isoform-specific antagonists of exchange proteins directly activated by cAMP. Proc Natl Acad Sci USA. 2012 109:18613-8. doi: 10.1073/pnas.1210209109.
3. Brown LM, Rogers KE, Aroonsakool N, McCammon JA, Insel PA. Allosteric inhibition of Epac: computational modeling and experimental validation to identify allosteric sites and inhibitors. J Biol Chem. 2014;289:29148-57. doi: 10.1074/jbc.M114.569319.
4. Chen H, Tsalkova T, Mei FC, Hu Y, Cheng X, Zhou J. 5-Cyano-6-oxo-1,6-dihydro-pyrimidines as potent antagonists targeting exchange proteins directly activated by cAMP. Bioorg Med Chem Lett. 2012;22:4038-43. doi: 10.1016/j.bmcl.2012.04.082. 22(12), 4038-4043.
5. Almahariq M, Tsalkova T, Mei FC, Chen H, Zhou J, Sastry SK, Schwede F, Cheng X. A novel EPAC-specific inhibitor suppresses pancreatic cancer cell migration and invasion. Mol Pharmacol. 2013;83:122-8. doi: 10.1124/mol.112.080689.
6. Juhász T, Matta C, Somogyi C, Katona É, Takács R, Soha RF, Szabó IA, Cserháti C, Sződy R, Karácsonyi Z, Bakó E, Gergely P, Zákány R. Mechanical loading stimulates chondrogenesis via the PKA/CREB-Sox9 and PP2A pathways in chicken micromass cultures. Cell Signal. 2014;26, 468-482. doi: 10.1016/j.cellsig.2013.12.001.

# Chen H, Wild C, Zhou X, Ye N, Cheng X, Zhou J. Recent advances in the discovery of small molecules targeting exchange proteins directly activated by cAMP (EPAC). J Med Chem. 2014;57:3651-65. doi: 10.1021/jm401425e.

# Ahmed A, Boulton S, Shao H, Akimoto M, Natarajan A, Cheng X, Melacini G. Recent advances in EPAC-targeted therapies: a biophysical perspective. Cells. 2019;8. pii: E1462. doi: 10.3390/cells8111462.

# Zhu Y, Chen H, Boulton S, Mei F, Ye N, Melacini G, Zhou J, Cheng X. Biochemical and pharmacological characterizations of ESI-09 based EPAC inhibitors: Defining the ESI-09 "therapeutic window". Sci.Rep. 2015:5:934. doi: 10.1038/srep09344.

1. Rehman H. Epac-ibhibitors: facts and artefacts. Sci Rep. 2013;3:3032.

**Supplementary Figure 3**


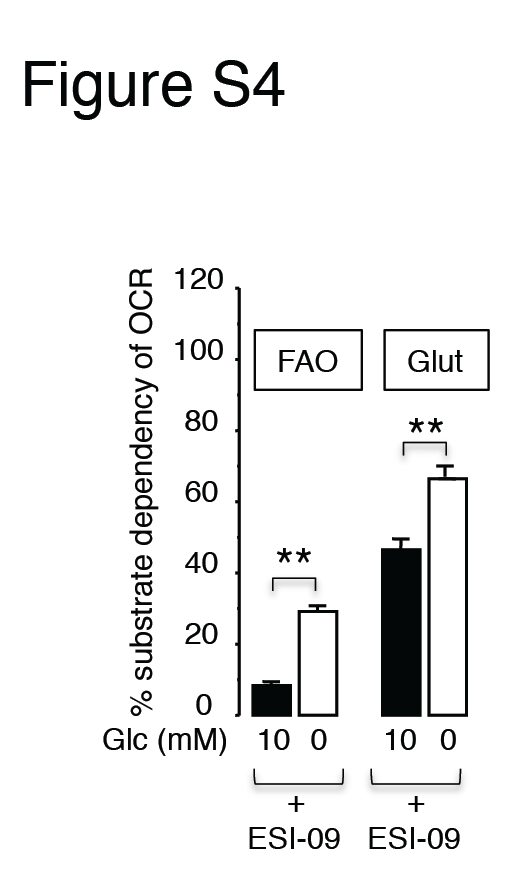


**Fig. S3**. Fuel oxidation dependency in A549 cells treated with ESI-09 (2 μM) in the presence or absence of glucose at pH 7.4. FAO, fatty acid oxidation; Glut, glutamine; Glc, glucose. ***P* < 0.01 by two-tailed unpaired *t* test (n = 3).

**Supplementary Figure 4**


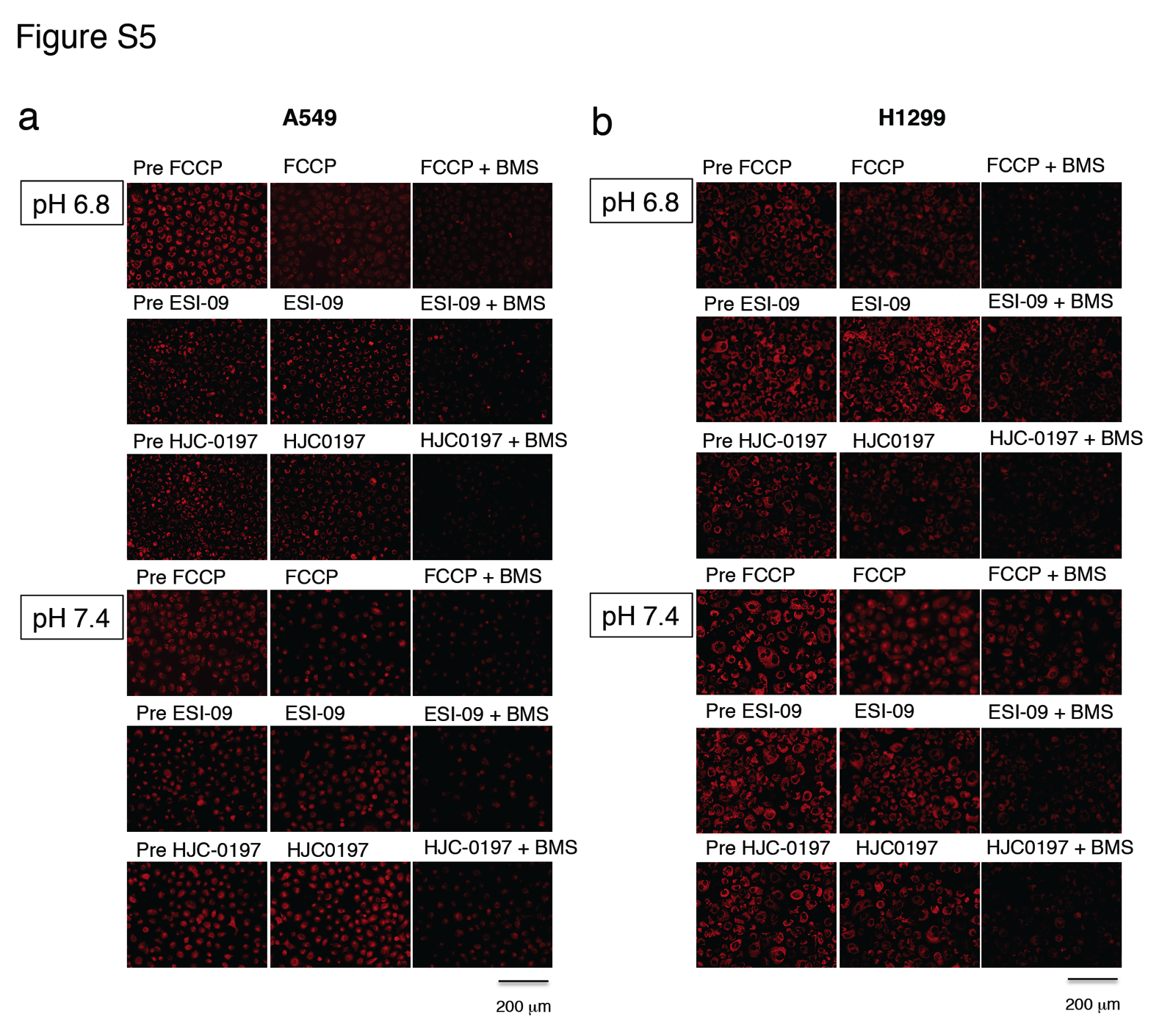


**Fig. S4**. Inhibition of F_1_F_0_ ATP hydrolase disrupts the maintenance of mitochondrial membrane potential (Δψm) in cells treated with mitochondrial uncouplers. A549 cells (**a**) or H1299 cells (**b**) were stained with tetramethylrhodamine methyl ester (250 nM) and treated with FCCP (2 μM), ESI-09 (2 μM), or HJC0197 (10 μM) in the presence or absence of BMS-199264 (2 μM), a selective mitochondrial F_1_F_0_ ATP hydrolase inhibitor. Results show a representative single result of three repetitions. FCCP, carbonyl cyanide 4-(trifluoromethoxy)phenylhydrazone; BMS, BMS-199264.

**Supplementary Figure 5**

**
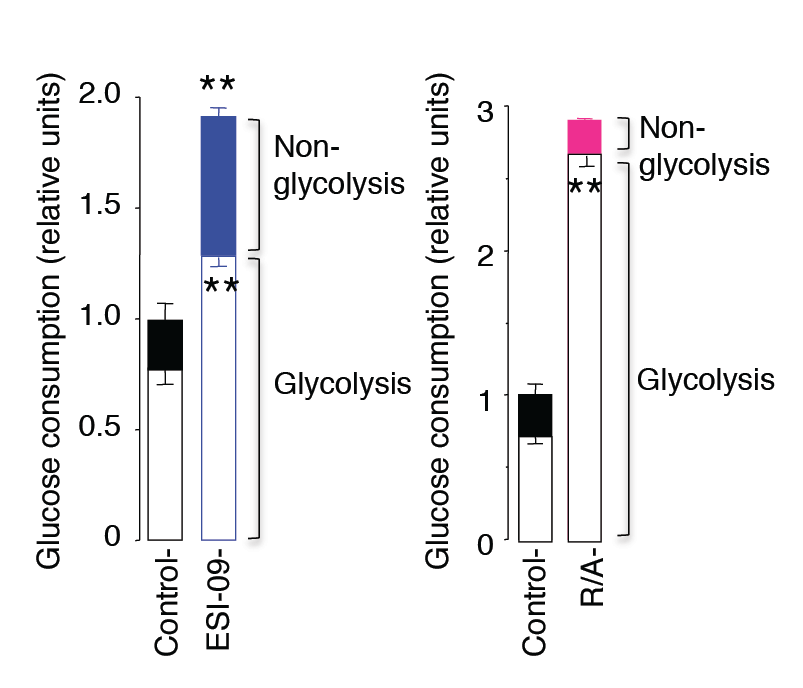
**

**Fig. S5**. The amount of glucose used for glycolysis and non-glycolysis (*i.e.*, tricarboxylic acid (TCA) cycle, *etc.*). Serum-starved confluent A549 cells were cultured in medium, pH 7.4, containing 15 mM glucose in the presence or absence of ESI-09 (2 μM) or the electron transport chain inhibitors rotenone (1 μM) plus antimycin A (1 μM). After 24 hours, the concentrations of glucose and lactate in medium were measured. The amount of glucose used for glycolysis was calculated by using the following formula based on the assumption that two molecules of lactate are produced during glycolysis of one molecule of glucose; the amount of glucose used for glycolysis (mol) = the amount of lactate production (mol) / 2. ***P* < 0.01 by two-tailed unpaired *t* test (n = 4).

**Supplementary Figure 6**


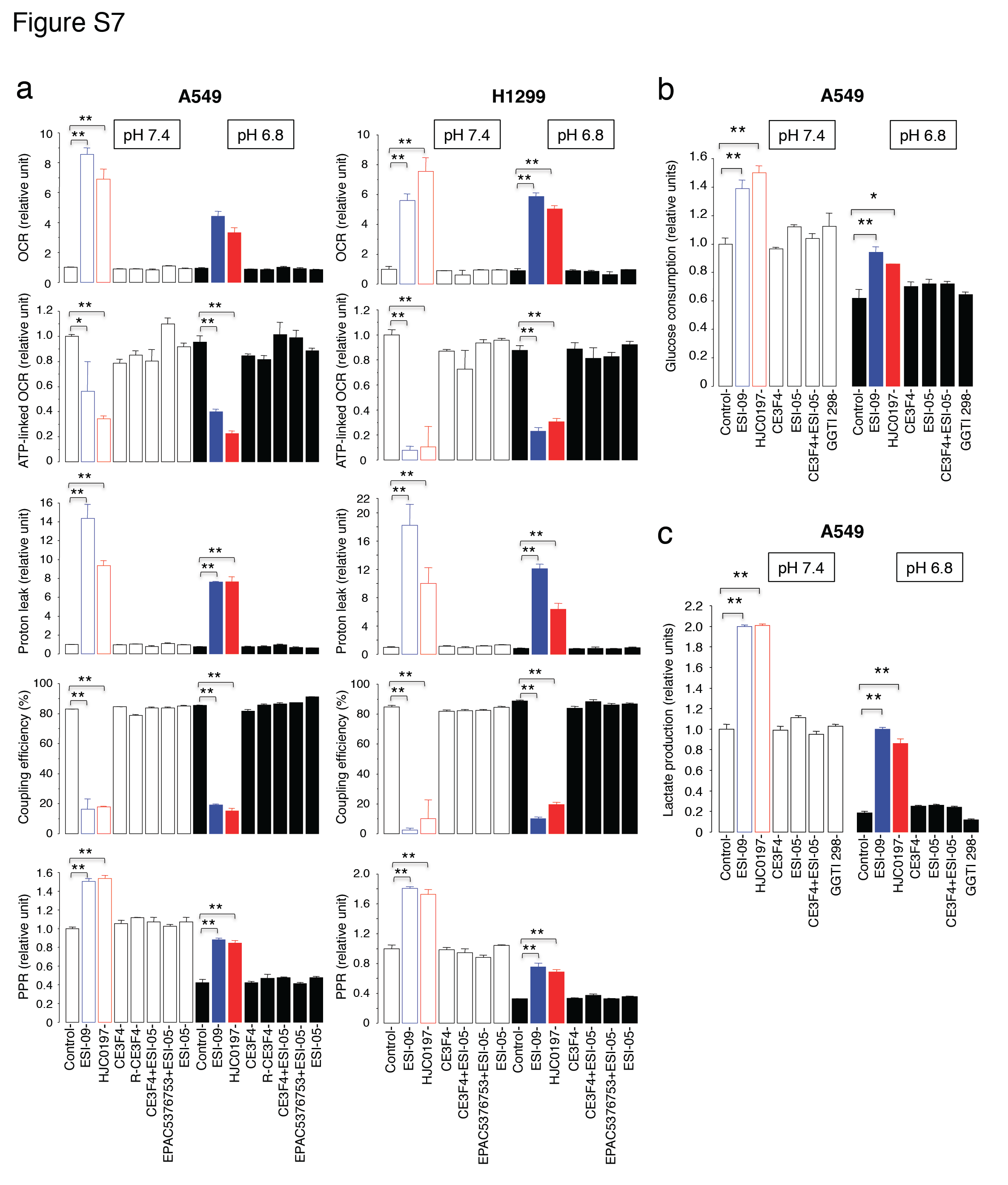


**Fig. S6**. Only ESI-09 and HJC0197 among various EPAC inhibitors show mitochondrial uncoupling activities. (**a**) Mitochondrial function measured with a Seahorse flux analyzer (n = 3 in each group). (**b**) Glucose consumption (n = 4). (**c**) Lactate production (n = 4). **P* < 0.05, ***P* < 0.01 by Dunnett’s test. OCR, oxygen consumption rate; PPP, proton production rate.

**Supplementary Figure 7**


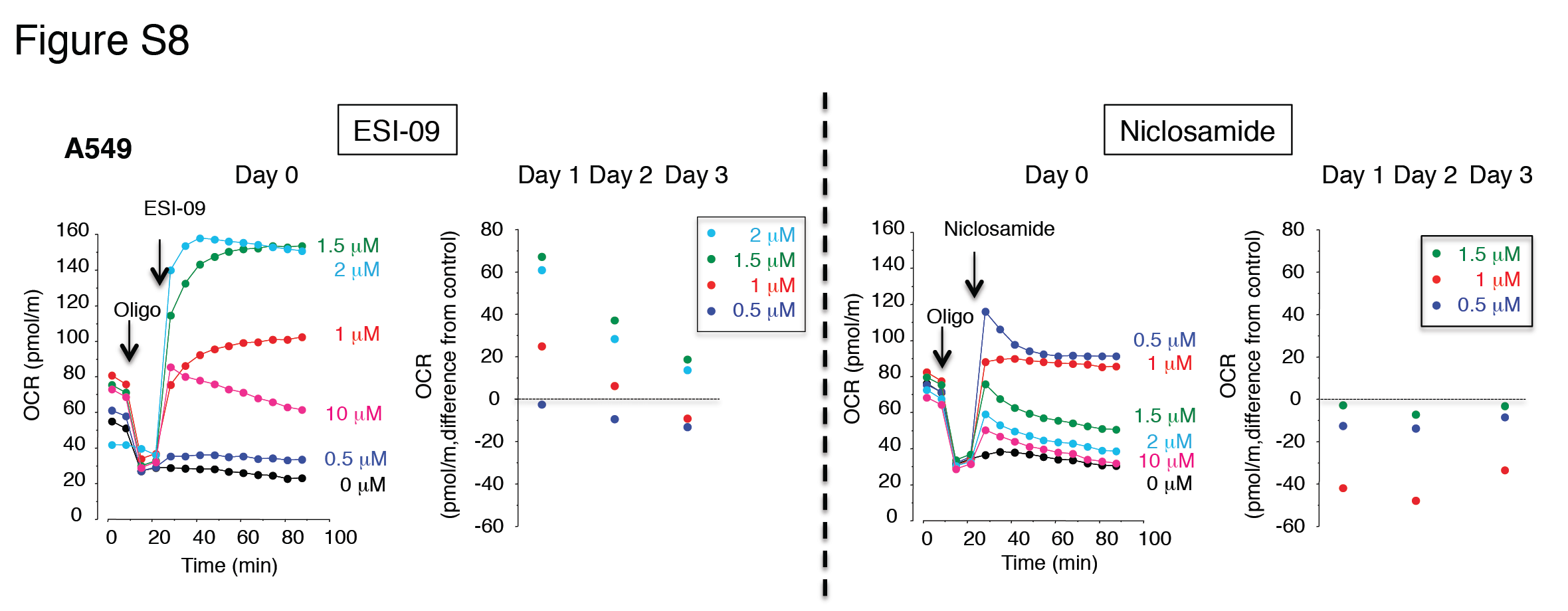
**Fig. S7**. Dose-dependent and time-course profile of the mitochondria uncoupling activities of ESI-09 and niclosamide. One representative dataset from two independent experiments is shown. Oligo, oligomycin; OCR, oxygen consumption rate.

**Supplementary Figure 8**


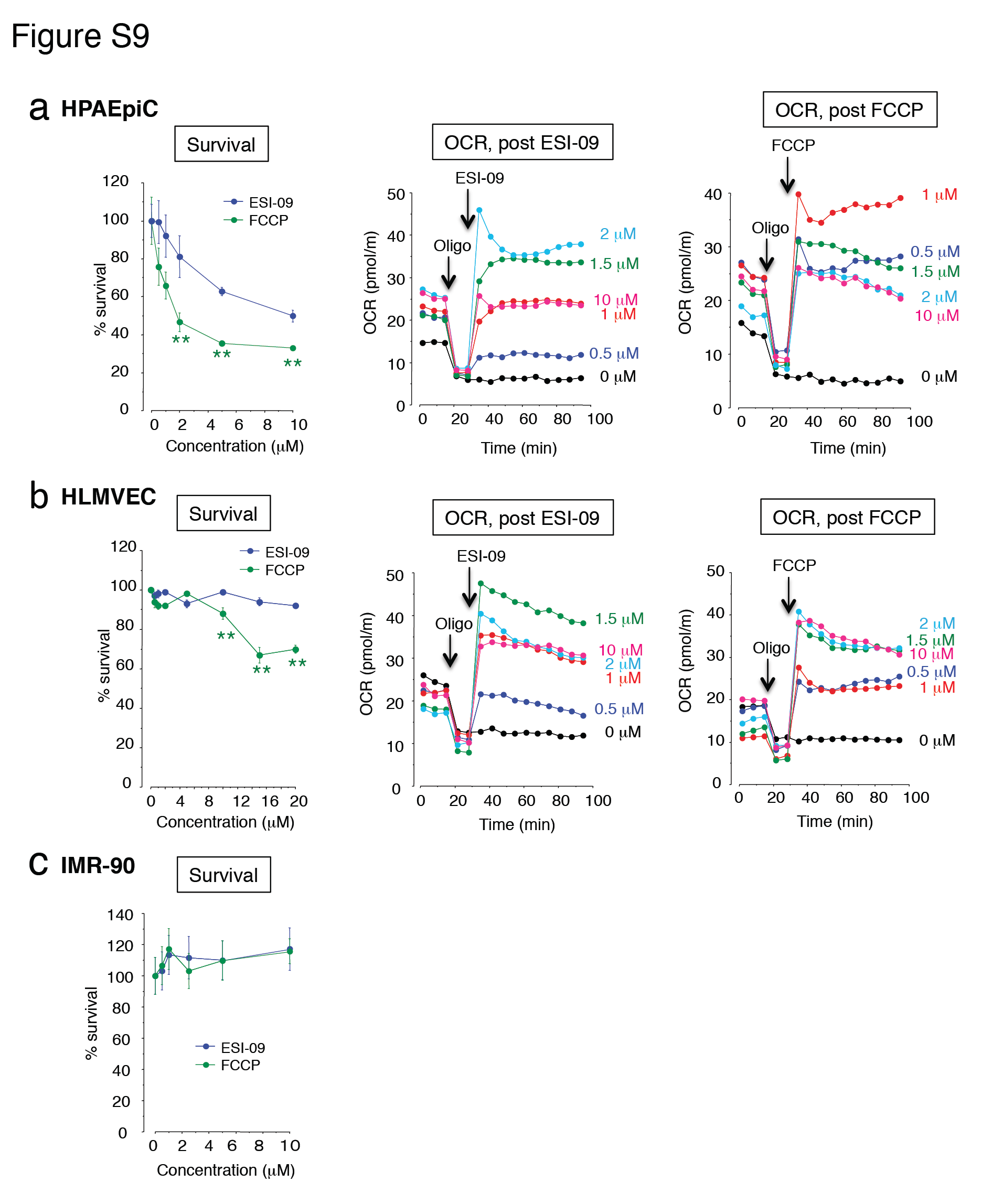


**Fig. S8.** Survival of non-cancerous cells treated with ESI-09 or FCCP. Confluent cultures of human pulmonary alveolar epithelial cells (HPAEpiC) (n = 8) (**a**), human lung microvascular endothelial cells (HLMVEC) (n = 8) (**b**) and fetal lung fibroblasts (IMR-90) (n = 8) (**c**) were treated for 72 hours with varying concentrations of FCCP or ESI-09 in culture medium, pH 7.4, containing 25 mM glucose. ***P* < 0.01 by two-tailed unpaired *t* test (n = 8). *Right panels*: dose-response effects of ESI-09 and FCCP on mitochondrial uncoupling. OCR, oxygen consumption rate; Oligo, oligomycin; FCCP, carbonyl cyanide 4-(trifluoromethoxy)phenylhydrazone.

**Supplementary Figure 9
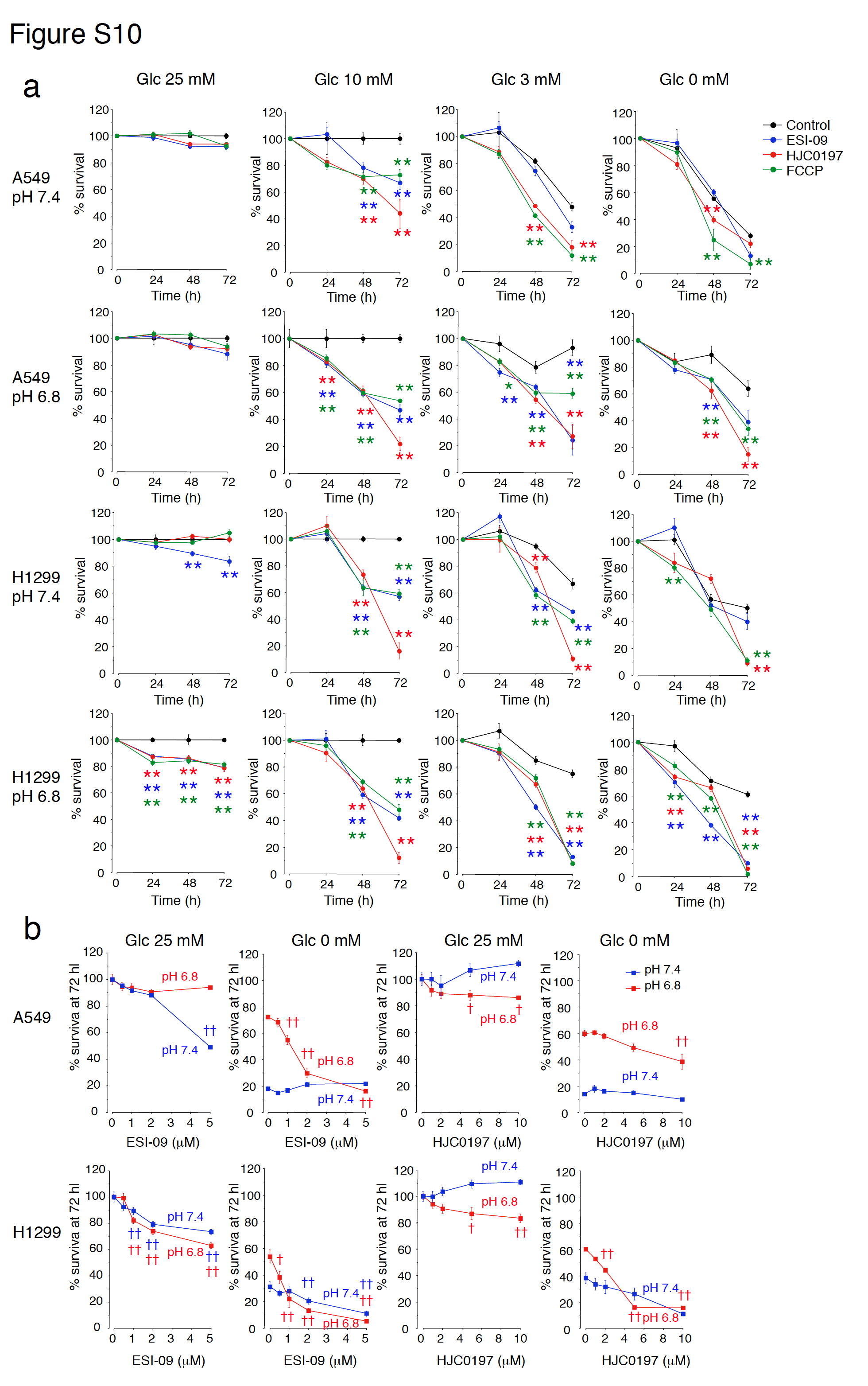
**

**Fig. S9.** Effects of mitochondrial uncouplers on the survival of A549 cells and H1299 cells cultured in medium, at pH 7.4 or pH 6.8, containing different initial concentrations of glucose. (**a**) Time-course effects of ESI-09 (2 μM), HJC0197 (10 μM), and FCCP (2 μM) on cell survival. **P* < 0.05, ***P* < 0.01 compared with control cells by Dunnett’s test (n = 8). (**b**) Dose–response effects of ESI-09 and HJC0197 on cell survival at 72 h. †*P* < 0.05, ††*P* < 0.01 by two-tailed unpaired *t* test when compared with untreated cells cultured in medium at pH 7.4 (*blue*) or at pH 6.8 (*red*) (n = 8). Glc, glucose; FCCP, carbonyl cyanide 4-(trifluoromethoxy)phenylhydrazone.

**Supplementary Figure 10**
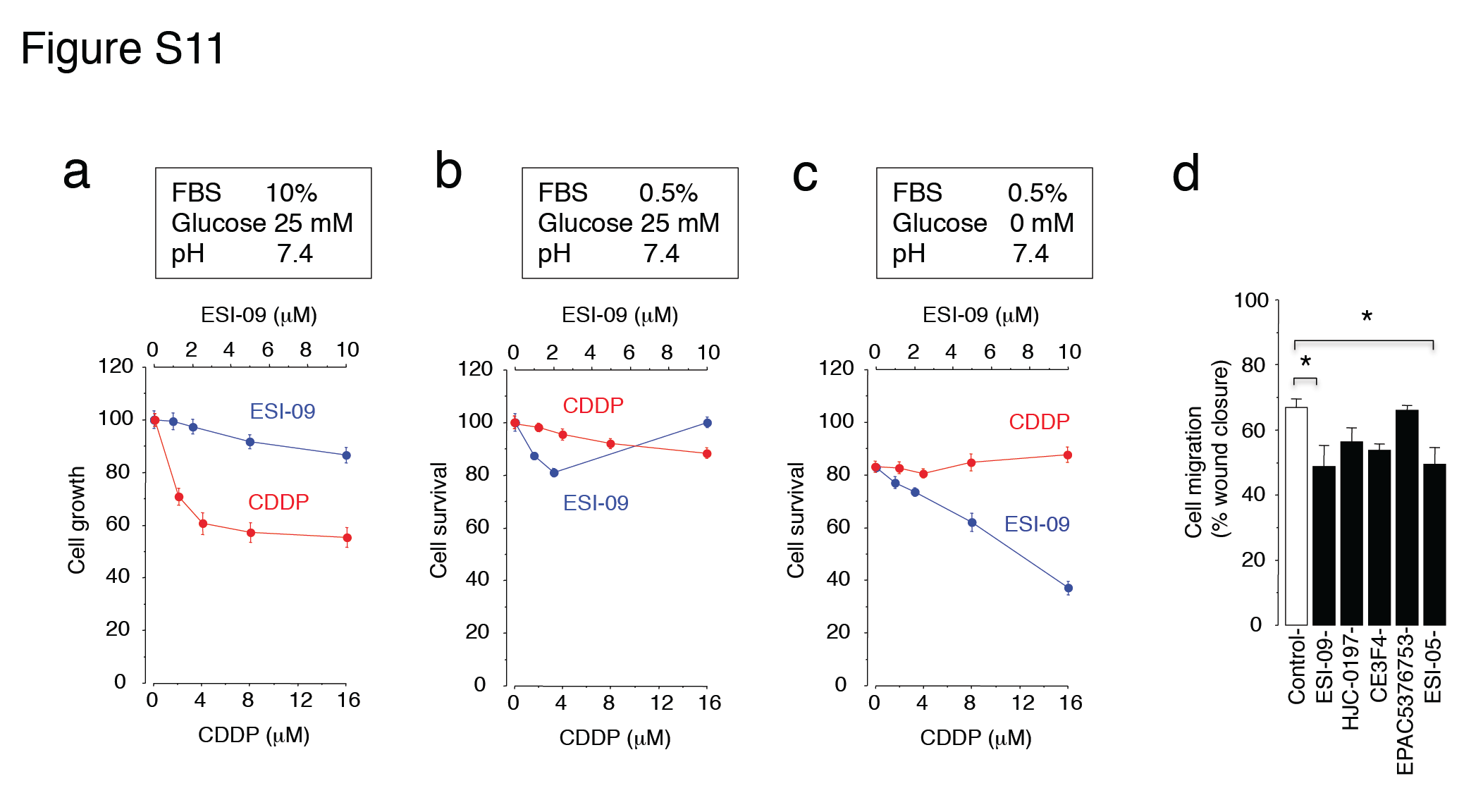


**Fig. S10.** Diverse effects of ESI-09 and cisplatin (CDDP) on A549 cells in a 2D monolayer culture. (**a**) Cell growth rate in full growth medium (25 mM glucose, 10% FBS, pH 7.4). (**b**) Survival rate of serum-starved confluent cells in medium containing glucose (25 mM glucose, 0.5% FBS, pH 7.4). (**c**) Survival rate of serum-starved confluent cells in medium lacking glucose (0 mM glucose, 0.5% FBS, pH 7.4). In **a** to **c**, A549 cells were incubated for 40 h in the indicated medium in the presence or absence of CDDP or ESI-09 (n = 8). (**d**) Effects of EPAC inhibitors on migratory activity of A549 cells. **P* < 0.01, ***P* < 0.01 by Dunnett’s test. (n = 4). FBS, fetal bovine serum.
